# Genes Related to Mitochondrial Functions, Protein Degradation, and Chromatin Folding Are Differentially Expressed in Lymphomonocytes of Rett Syndrome Patients

**DOI:** 10.1155/2013/137629

**Published:** 2013-12-12

**Authors:** Alessandra Pecorelli, Guido Leoni, Franco Cervellati, Raffaella Canali, Cinzia Signorini, Silvia Leoncini, Alessio Cortelazzo, Claudio De Felice, Lucia Ciccoli, Joussef Hayek, Giuseppe Valacchi

**Affiliations:** ^1^Department of Molecular and Developmental Medicine, University of Siena, 53100 Siena, Italy; ^2^Child Neuropsychiatry Unit, University Hospital, Azienda Ospedaliera Universitaria Senese, 53100 Siena, Italy; ^3^National Research Institute on Food and Nutrition (INRAN), 00178 Rome, Italy; ^4^Department of Life Science and Biotechnologies, University of Ferrara, 44121 Ferrara, Italy; ^5^Department of Medical Biotechnologies, University of Siena, 53100 Siena, Italy; ^6^Neonatal Intensive Care Unit, University Hospital, Azienda Ospedaliera Universitaria Senese, 53100 Siena, Italy; ^7^Department of Food and Nutrition, Kyung Hee University, Seoul 130-701, Republic of Korea

## Abstract

Rett syndrome (RTT) is mainly caused by mutations in the X-linked methyl-CpG binding protein (*MeCP2*) gene. By binding to methylated promoters on CpG islands, MeCP2 protein is able to modulate several genes and important cellular pathways. Therefore, mutations in *MeCP2* can seriously affect the cellular phenotype. Today, the pathways that *MeCP2* mutations are able to affect in RTT are not clear yet. The aim of our study was to investigate the gene expression profiles in peripheral blood lymphomonocytes (PBMC) isolated from RTT patients to try to evidence new genes and new pathways that are involved in RTT pathophysiology. LIMMA (Linear Models for MicroArray) and SAM (Significance Analysis of Microarrays) analyses on microarray data from 12 RTT patients and 7 control subjects identified 482 genes modulated in RTT, of which 430 were upregulated and 52 were downregulated. Functional clustering of a total of 146 genes in RTT identified key biological pathways related to mitochondrial function and organization, cellular ubiquitination and proteosome degradation, RNA processing, and chromatin folding. Our microarray data reveal an overexpression of genes involved in ATP synthesis suggesting altered energy requirement that parallels with increased activities of protein degradation. In conclusion, these findings suggest that mitochondrial-ATP-proteasome functions are likely to be involved in RTT clinical features.

## 1. Introduction

Rett syndrome (RTT) is a rare form of autism spectrum disorder (ASD), which mostly affects girls with worldwide prevalence rate ranges from 1 : 10,000 to 1 : 20,000 live births [1–5]. RTT is a clinically defined condition with a large spectrum of phenotypes associated with a wide genotypic variability [6, 7]. Classic or typical RTT, the most common form of the condition, is caused in about 90–95% of cases by *de novo* mutations in the *MeCP2*, a gene mapped on chromosome X and encoding methyl-CpG binding protein 2 [7, 8]. The clinical picture of classical form progresses through 4 stages and is characterized by normal development for the first 6 to 18 months, followed by loss of purposeful hand movements, failure of speech development, autistic-like behavior, slowed brain and head growth, and mental retardation [9].

To date, it is not known how *MeCP2* mutations lead to RTT phenotypes; therefore the identification of the pathways that are affected by *MeCP2 *functions could bring new insight in the RTT pathogenetic mechanisms. MeCP2 was originally thought to function as a transcription repressor by binding to methylated CpG dinucleotides, but recent studies have individuated more functions related to MeCP2 [10, 11]. In fact MeCP2 is now considered a multifunctional protein, since it is implicated not only in genome transcriptional silencing, but also in transcriptional activation, by regulating chromatin and nuclear architecture [11]; therefore, its malfunction or mutation can lead to severe cellular function alterations.

Hence, it is very difficult to understand the link between *MeCP2* mutation and the clinical feature present in RTT. One of the most common approaches used to better understand the molecular pathways involved in genetic disorders has been the determination of gene expression profiling, since it provides the opportunity to evaluate possible transcriptome alterations at both gene and gene-network levels. This approach should not be considered an end point but a magnifying lent where new aspects involved in the diseases can be discovered and then studied.

So far, only a handful of studies have investigated the gene expression profiles of RTT children in tissues, that is, postmortem brain samples [12], or in cells, such as clones of fibroblasts isolated from skin biopsies [13, 14] and immortalized lymphoblastoid cell lines [14–16]. Moreover, several studies have performed microarray gene expression analysis using *in vitro* cellular models representing MeCP2 deficiency induced by siRNAs [17] or cells and tissues from RTT mouse models [18], but to our knowledge there are no data on microarray analysis from “*ex vivo*” fresh samples. For this reason, the aim of this study was to evaluate the gene expression patterns in PBMC isolated from RTT patients. This approach lets us bypass some of the limitations/variables of the previous gene arrays studies on RTT, such as the use of postmortem samples, gene-modified cells and murine tissues that do not always reflect all features of the human disease. In fact, studying *ex vivo* samples, such as PBMC, provides some advantage that can be summarized by the fact PBMC are the only readily available cells in humans; various studies showed disease-characteristic gene expression patterns in PBMC that can be easily obtained.

Our results identified a clear difference in gene expression profile between control and RTT patients, with almost 500 genes being deregulated, suggesting several new pathways involved in this disorder.

## 2. Subjects and Methods

### 2.1. Subjects Population

The study included 12 female patients with clinical diagnosis of typical RTT (mean age: 10.9 ± 4.9 years, range: 6–22) with demonstrated *MeCP2* gene mutation and 7 sex- and age-matched healthy controls (mean age: 15.1 ± 9.03 years, range: 4–32). RTT diagnosis and inclusion/exclusion criteria were based on the recently revised RTT nomenclature consensus [4]. All the patients were consecutively admitted to the Rett Syndrome National Reference Centre of the University Hospital of the Azienda Ospedaliera Universitaria Senese (AOUS).  Table 1 presents the demographic and genetic characteristics of the enrolled patients subjected to microarray analysis. Blood sampling in the control group was carried out during routine health checks, sports, or blood donations, while blood sample in patients were obtained during the periodic clinical checks. The study was conducted with the approval of the Institutional Review Board and all informed consents were obtained from either the parents or the legal tutors of the enrolled patients.

### 2.2. Blood Specimen Collection, Peripheral Blood Lymphomonocytes Isolation, and RNA Extraction

Blood was collected in heparinized tubes and all manipulations were carried out within 30 minutes after sample collection. PBMC were separated from whole blood by density gradient centrifugation using Ficoll-Paque PLUS (GE Healthcare Europe GmbH, Milan, Italy). After PBMC isolation, total RNA was extracted from cells using RNeasy mini kit (Qiagen, Hilden, Germany), according to the manufacturer's instructions. The total nucleic acid concentration and purity were estimated using a NanoDrop spectrophotometer (NanoDrop Technologies, Wilmington, DE). Quality of RNA was checked on Agilent bioanalyzer (Agilent Technologies, Santa Clara, CA). The isolated RNA samples were stored at −80°C until the analysis.

### 2.3. Microarray Processing

For the microarray processing, RNA was amplified and labeled using the Affymetrix Whole-Transcript (WT) Sense Target Labeling Protocol. Affymetrix GeneChip Human Gene 1.0 ST arrays were hybridized with labeled sense DNA, washed, stained, and scanned according to the protocol described in WT Sense Target Labeling Assay Manual.

Briefly, 100 ng of total RNA was reverse transcribed into double-stranded cDNA with random hexamers tagged with a T7 promoter sequence. The double-stranded cDNA was subsequently used as a template and amplified by T7 RNA polymerase, producing many copies of antisense cRNA. In the second cycle of cDNA synthesis, random hexamers were used to prime reverse transcription of the cRNA from the first cycle to produce single-stranded DNA in the sense orientation. dUTP was incorporated in the DNA during the second-cycle, first-strand reverse transcription reaction. This single-stranded DNA sample was then treated with a combination of uracil DNA glycosylase (UDG) and apurinic/apyrimidinic endonuclease 1 (APE 1) that specifically recognized the unnatural dUTP residues and broke the DNA strand. DNA was labeled by terminal deoxynucleotidyl transferase (TdT) with the Affymetrix proprietary DNA Labeling Reagent that is covalently linked to biotin. 5 *μ*g of labeled cDNA was hybridized to the Human Gene 1.0 ST Array at 45°C for 17 hours. The arrays were washed and stained in Affymetrix Fluidics Station 450 and scanned using the Affymetrix GeneChip Scanner 3000.

### 2.4. Microarray Data Analysis

The microarray experiments were analyzed with the oneChannelGUI package available in R software. The signal intensities from each chip were preliminarily normalized with RMA method and filtered by IQR filter choosing as threshold a value of 0.25. This specific filter removes the probesets that do not present changes in their expression across the analyzed microarray. The threshold of 0.25 is an intermediate value that retains the probesets that show significative changes of their signal at least in the 25% of analyzed microarray. The microarray data were analyzed by SAM and LIMMA analyses. SAM approach uses permutation based statistics and is a valid method to analyze data that may not follow a normal distribution, outperforming other techniques (e.g., ANOVA and classical *t*-test), which assume equal variance and/or independence of genes. LIMMA fit each gene expression to a linear regression model testing the significativity of the distance from the model with a *t*-test robust against nonnormality and inequality of variances. In order to characterize the biological processes enriched in the list of modulated genes, a statistical analysis of overrepresented Gene Ontology (GO) terms (BP level 5) was performed with DAVID (Database for Annotation, Visualization, and Integrated Discovery) web server (DAVID Bioinformatics Resources) [19]. Only the GO terms enriched with a *P* value < 0.05 corrected with the Benjamini and Hochberg method [20] were selected and hierarchically clusterized (Ward's method) according to their “semantic” similarity estimated with the GOSIM R package with default parameters. After clusterization the best number of clusters was identified according to the analysis of the silhouette scores and the medoid term in each cluster was selected as the representative member of the cluster. Each cluster represents a group of biological processes with similar e/o related functions.

### 2.5. Validation of Microarray Data by RT-qPCR (Reverse Transcription Quantitative Real-Time PCR)

For confirmation of Affymetrix expression microarray results, RT-qPCR analysis was performed as previously described [21]. For validation, six of the differentially expressed genes, 3 upregulated—*GSTO1*, *PSMB10* and *COX8A*—and 3 downregulated—*HIST1H1B*, *MMP9* and *ARHGAP11B*—by microarray, were chosen. Validation was done in a randomly selected subset of the original samples (submitted for microarray analysis) that included 3 healthy controls and 3 RTT patients. Primer pairs were obtained from the Real-time PCR GenBank Primer to hybridise unique regions of the appropriate gene sequence: *GSTO1* (Fw: 5′-AGA GTT GTT TTC TAA GGT TCT GAC T-3′) and (RW: 5′-ACT TCA TTG CTT CCA GCC GT-3′), product length 116 bp; *PSMB10* (Fw: 5′-ACA GAC GTG AAG CCT AGC AG-3′) and (Rw: 5′-ACC GAA TCG TTA GTG GCT CG-3′), product length 294 bp; *COX8A* (Fw: 5′-GCC AAG ATC CAT TCG TTG CC-3′) and (RW: 5′-TCT GGC CTC CTG TAG GTC TC-3′), product length 137 bp; *HIST1H1B* (Fw: 5′-CCC GGC TAA GAA GAA GGC AA-3′) and (RW: 5′-ACA GCC TTG GTG ATC AGC TC-3′), product length 99 bp; *MMP9* (Fw: 5′-GTC CGT GAG GGT GTT GAG TG-3′) and (RW: 5′-ACT GCT CAA AGC CTC CAC AA-3′), product length 145 bp; *ARHGAP11B *(Fw: 5′-AAC TGC CAG AGC CCA TTC TC-3′) and (RW: 5′-GTC TGG TAC ACG CCC TTC TT-3′), product length 295 bp. All reactions were run in triplicate. *GAPDH* (FW: 5′-TGA CGC TGG GGC TGG CAT TG-3′ and RW: 5′-GGC TGG TGG TCC AGG GGT CT-3′, 134 pb) was used in our experiments as internal standard. As previously described, samples were compared using the relative cycle threshold (CT) method (Livak and Schmittgen 2001). After normalization to more stable mRNA GAPDH, the fold increase or decrease was determined with respect to control, using the formula 2^−ΔΔCT^, where ΔCT is (gene of interest CT)−(reference gene CT) and ΔΔCT is (ΔCT experimental)−(ΔCT control). Results are the means ± SEM of three independent experiments, each analysed in triplicate. *P < 0.001 versus control (one-way ANOVA followed by Bonferroni's posttest).

## 3. Results and Discussion

### 3.1. Differentially Regulated Genes in RTT Patients

Given that RTT results from dysfunction of the transcriptional modulator MeCP2, several strategies have been developed to identify its target genes in order to gain insights into the disease pathogenesis [12–18]. In our study, to identify gene expression changes associated with and potentially related to *MeCP2* mutations and to delineate alterations of pathways associated with the disease, we evaluated and compared transcriptomic profiles in PBMC from RTT patients and control subjects.

This work showed for the first time, to our knowledge, altered expression of a large set of genes that may help elucidating and explaining the link between MeCP2 and some of the molecular and cellular aspects observed in RTT patients. In our previous studies on RTT we were able to show increased levels of oxidative stress (OS) markers, such as isoprostanes (IsoPs) and 4-hydroxynonenal protein adducts (4-HNE PAs) [22, 23], and increased ubiquitination and degradation of oxidatively modified proteins [24], but how *MeCP2* mutation is able to affect cellular redox balance and proteins turn over still needs to be defined.

**Figure 1:**
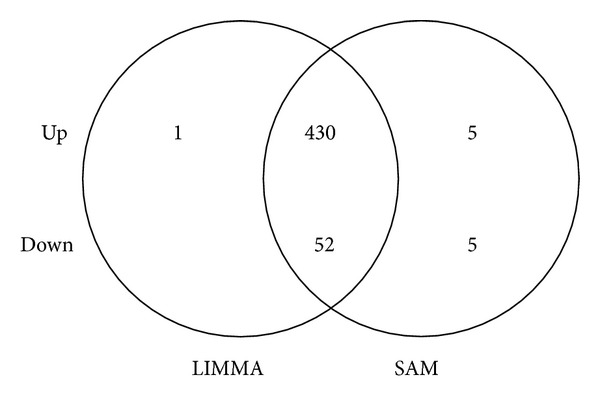
Comparison of two distinct approaches for screening of differentially expressed genes in RTT PBMC. Venn diagram representing numbers of common and exclusively up- and downregulated genes for LIMMA (left) and SAM (right) analyses (FC ± 1; adj. *P* value ≤ 0.05). The 11 genes indicated by only one method were excluded by further analysis.

Using the SAM and LIMMA methods, we have defined a set of genes differentially expressed in RTT patients with respect to controls (healthy subjects) and a significant overlap was found by comparing results from two approaches. A cut-off level based on a minimum of 1-fold change in expression resulted in a list of 482 common deregulated genes, while 10 genes were suggested only by SAM and only 1 gene was indicated by LIMMA. Among the shared genes, 430 showed significant upregulation, while 52 were downregulated in RTT compared to controls (Figure 1 and Supplemental Tables  1 and  2). The 11 genes indicated by only one method were excluded for further analysis. Genes with the strongest changes in expression, both upregulated (FC ≥ 2) and downregulated (FC ≤ −1.2), are listed in Tables 2 and 3, respectively. The complete list of differentially expressed genes, both upregulated and downregulated (FC ± 1), is shown in Supplemental Tables  1 and  2.

It is evident from this first set of data that mutations in *MeCP2* influence more the genes upregulation with respect to the downregulation. This is in part in line with the first functions that have been attributed to MeCP2, such as a gene expression repressor [10, 11]. In addition, it is worth it to underline that both screening approaches used in this study (LIMMA and SAM) almost overlap between them making the results more reliable.

Next, using the DAVID databases, we performed the functional annotation of the significant genes and identification of the biochemical pathways in which they are involved. A comparison of the differentially regulated mRNA transcripts in RTT PBMC compared to control group shows significant changes in cellular pathways. In particular, we identify 10 major clusters corresponding to 62 biological processes enriched by 146 genes (Table 4). These clusters highlight key biological pathways related to mitochondrial function and organization (i.e., mitochondrial ATP synthesis coupled to electron transport, inner mitochondrial membrane organization such as: *ATP5A1*, *COX6C*, *ETFA*, *UQCRQ*, *TIMM10*, and *TSPO*), cellular protein metabolic process (i.e., regulation of protein ubiquitination, regulation of ubiquitin-protein ligase activity, and proteasomal ubiquitin-dependent protein catabolic process such as *PSMA2*, *PSMD6*, *UBE2E3*, and *UFC*), RNA processing (i.e., nuclear mRNA splicing and spliceosome assembly and RNA elongation from RNA polymerase II promoter such as *RPL15*), DNA organization in chromatin, and cellular macromolecular complex assembly (i.e., nucleosome assembly, DNA packaging, and protein complex biogenesis such as *HIST1H4L*, *H2AFZ*, *TOP2A*, and *HMGB2*).

### 3.2. Mitochondrial Related Genes in RTT Patients

Among these clusters, the most significantly regulated transcripts include those encoding several subunits of mitochondrial respiratory chain complexes and thus linked directly to mitochondrial ATP production and, indirectly, to potential reactive oxygen species (ROS) generation. In particular, *NDUFA1*, *NDUFAB1*, *NDUFA2*, and *NDUFB6*, all components of mitochondrial complex I (NADH: ubiquinone oxidoreductase), showed the greater changes with a FC of more than 2. Moreover, other subunits of complex I (*NDUFV2*, *NDUFS4*, *NDUFA9*, *NDUFS6*, *NDUFB10*, *NDUFB4*, *NDUFC2*, *NDUFB2*, *NDUFS5*, *NDUFC1*, *NDUFB9*, and *NDUFA8*) were clearly upregulated in RTT group.

Complex I plays a vital role in cellular ATP production, the primary source of energy for many crucial processes in living cells. It removes electrons from NADH and passes them by a series of different protein coupled redox centers to the electron acceptor ubiquinone. Because complex I is central to energy production in the cell, it is reported that its malfunction results in a wide range of neuromuscular diseases [25]. Some of them are due to mutations in the mitochondrial genome, but others, which result from a decrease in the activity of complex I or an increase in the production of ROS, are not well understood. The production of ROS by complex I is linked to Parkinson's disease and to ageing [26, 27] and this is in line with RTT since it is now well documented as an increased OS condition in this pathology [22, 23].

Another gene involved in complex I function is *NDUFV2* that was also clearly upregulated in our study. Mutations in this gene are implicated in Parkinson's disease, bipolar disorder, and schizophrenia and have been found in one case of early onset hypertrophic cardiomyopathy and encephalopathy; also it has been shown for *NDUFA2*, a subunit of the hydrophobic protein fraction of the complex I. Mutations in this gene are associated with Leigh syndrome, an early onset progressive neurodegenerative disorder. Of note is *NDUFAB1*, which is a carrier of the growing fatty acid chain in fatty acid biosynthesis in mitochondria and alteration in fatty acid levels has been noted in ASD [28, 29].

Not only complex I subunits were upregulated in RTT, but also we have detected an upregulation of genes involved in all the five complexes of the electron transport chain. In fact, also *SDHB* (succinate dehydrogenase complex, subunit B, and iron sulfur (Ip)) gene encoding for a subunit of mitochondrial complex II (succinate: ubiquinone oxidoreductase) was significantly upregulated. This subunit is responsible for transferring electrons from succinate to ubiquinone (coenzyme Q). Complex II of the respiratory chain, which is specifically involved in the oxidation of succinate, carries electrons from FADH to CoQ. Of note, also 3 genes, *UQCRQ*, *UQCRFS1,* and *UQCRH*, encoding for subunits of complex III (ubiquinol-cytochrome c oxidoreductase), were upregulated with a mean FC = 1.60. This complex plays a critical role in biochemical generation of ATP, contributing to the generation of electrochemical potential by catalyzing the electron transfer reaction from ubiquinol to cytochrome c coupled with proton translocation across the membrane. Lines of evidence report that in mouse models some of the promoters of ubiquinol-cytochrome c reductase subunit are able to be targeted by MECP2 protein, contributing to the development of the pathology [30].

Furthermore, the cytochrome c gene (*CYCS*) together with genes encoding subunits of mitochondrial complex IV (cytochrome c oxidase) (*COX14*, *COX7A2*, *COX6C*, *COX7C*, and *COX8A*) was upregulated with a mean FC of circa 1.5. Of note is the upregulation of cytochrome c gene. The encoded protein accepts electrons from cytochrome b and transfers them to the cytochrome oxidase complex. This protein is also involved in initiation of apoptosis and this would be in line with previous studies that have shown a possible involvement of apoptosis in RTT [31, 32], although only few studies have investigated the role of apoptosis in RTT and the current literature is still controversial. In addition, several subunits of complex IV were upregulated. It receives an electron from each of the four cytochrome c molecules and transfers them to one oxygen molecule, converting molecular oxygen to two molecules of water. In the process, via a protons translocation, it is able to generate ATP. This data would suggest that RTT patients are in continuous new ATP synthesis and this could be a consequence of new protein synthesis.

Our data are in line with a previous work by Kriaucionis where the authors have analyzed the gene profile in the brain of RTT animal model [30]. They have shown that there were several mitochondrial abnormalities and an upregulation of both complexes I and III subunits. In particular they were also able to show a correlation between upregulation of complexes I and III and the animal symptoms severity with a significant increase in mitochondrial respiratory capacity and a reduction in respiratory efficiency. The defect appears to be associated with respiratory complex III, which is also upregulated in our study, and that containing the Uqcrc1 protein. In addition it has been shown that MeCP2 binds to the promoter of the Uqcr gene *in vivo* and that Uqcr mRNA expression is elevated in brains of *Mecp2*-null mice that have acquired neurological symptoms and this is in line with our results.

Finally, we also observed in RTT PBMC an upregulation of mitochondrial complex V (ATP synthase) subunits (*ATP5A1*, *ATP5EP2*, *ATP5J2*, and *ATP5O*) together with ATPase inhibitory factor 1 gene (*ATPIF1*). Mitochondrial membrane ATP synthase is a master regulator of energy metabolism and cell fate; therefore, a misregulation of this gene can be associated with altered ATP production and cell metabolism. It is interesting to note that also the ATPase inhibitory factor 1 (*ATPIF1*) that inhibits the activity of the mitochondrial H^+^-ATP synthase was upregulated, and this lets us speculate the existence of an aberrant loop between making new ATP and inhibiting its production. In addition, recent findings indicate that *ATPIF1* has additional functions by promoting adaptive responses of cell to ROS [33], a condition (OS) that has been well documented to be present in RTT [22, 23].

Similarly, other genes related to the ATP synthesis showed significant expression changes (i.e., *CYB5A*, *CYB561D2*, *ETFA*, *LDHB*, *PDHB,* and *SURF1*). For instance, *ETFA* (electron transfer flavoprotein, alpha polypeptide) serves as a specific electron acceptor for several dehydrogenases and in mitochondria it shuttles electrons between primary flavoprotein dehydrogenases and the membrane-bound electron transfer flavoprotein ubiquinone oxidoreductase. In addition, *LDHB* encodes lactate dehydrogenase B, an enzyme that catalyzes the reversible conversion of lactate and pyruvate and NAD and NADH, in the glycolytic pathway, being therefore correlated with ATP generation. Of note is the upregulation of *SURF1* (surfeit 1) gene that encodes a protein localized to the inner mitochondrial membrane and thought to be involved in the biogenesis of the cytochrome c oxidase complex.

Related to mitochondrial structure/organization, we found upregulation of 7 translocase genes (*TIMM10*, *TSPO*, *TIMM9*, *TIMM17A*, *TOMM7*, *TIMM13,* and *TIMM8B*) involved in proteins import into mitochondrion (mean FC of 1.34) and of several mitochondrial ribosomal proteins (*MRPL13*, *MRPL20*, *MRPL21*, *MRPL33*, *MRPL51*, *MRPL52*, *MRPS25*, *MRPS30*, *MRPS33*, *MRPS36*, *RPL10A*, *RPL13*, *RPL15*, *RPL22*, *RPL22L1*, *RPL26*, *RPL31*, *RPL32*, *RPL39L*, *RPS10*, *RPS25*, *RPS26*, *RPS26P11*, *RPS29*, *RPS5,* and *RPS7*) with a mean FC of 1.5.

All together these lines of evidence seem to suggest an increased mitochondrial activity that might be linked to the observation of the pathologic phenotype. Anyways at this stage of the study, it is not possible to determine whether or not there is an increase in ATP levels. It is possible to speculate that increased genes related to mitochondrial subunits could be a consequence of increased cells request of energy (ATP). This hypothesis is in line with recent study where the authors have shown decreased levels of ATP in brain mouse RTT [34].

Recent discussions regarding a possible connection between RTT and mitochondrial dysfunction have generated significant interest. The basis for these discussions is related in part to the common features of RTT on the one hand and disorders of mitochondrial function on the other. Of interest is the fact that a patient with symptoms normally associated with mitochondrial disorders harbored mutations in the *MeCP2* gene [35]. This overlap between symptoms of RTT and mitochondrial disorders recalls early reports of structural abnormalities [36, 37] and defects in the electron transport chain [37, 38] in mitochondria from skin and muscle biopsies of RTT patients. Moreover, about half of RTT patients were reported to have elevated levels of circulatory lactic or pyruvic acid, which might be caused by defects in the efficiency of the respiratory chain and urea cycle complexes, both of which are mitochondrial [39–41]. Several disorders related to the brain are a consequence of mitochondrial alteration with the resultant increase of OS and in certain cases the cell apoptosis. As RTT is not a neurodegenerative disorder [42], any contribution of mitochondrial dysfunction to RTT symptoms may take the form of chronic mitochondrial underperformance, rather than catastrophic failure leading to neuronal death.

To date, no systematic study of mitochondrial function in individuals with RTT has presented whether these findings represent a primary or secondary effect; that is, are they involved directly in the clinical features of RTT or do they reflect effects of these clinical features on mitochondrial function? Prior to identification of mutations in *MeCP2* in 1999, several reports appeared to be related to mitochondrial structure and function [36–54]. However, since 2001, publications on a possible role of mitochondria in the pathogenesis of RTT have been very few [36, 54]. In a recent work, investigators in Australia reported gene expression results from postmortem brain tissue of individuals with RTT and normal controls [55]. One gene related to a mitochondrial enzyme, cytochrome c oxidase subunit 1, had reduced expression in RTT tissue raising the possibility that loss of MeCP2 function could be responsible. However, whether this is a primary or secondary finding remains to be established and provides an important target for further investigation.

In summary, while mitochondrial abnormalities related to structure and functions have been reported, sufficient information is lacking as to the precise role of such abnormalities in RTT. As mentioned, alteration of mitochondrial functions is often correlated with OS and, in particular, the mitochondrial sites that are often invoked as the most important mitochondrial superoxide producers are in respiratory complexes I and III [56, 57] and this can explain the increased OS levels found in RTT patients.

### 3.3. Oxidative Stress Related Genes in RTT Patients

The presence of a redox unbalance in RTT is confirmed by the upregulations of several genes involved in redox homeostasis such as superoxide dismutase 1, catalase, and peroxiredoxin 1 (*SOD1*, *CAT*, and *PRDX1*) with a 1.6, 1.14, and 1.12 FC, respectively. Moreover, glutathione S-transferase omega 1, microsomal glutathione S-transferase 2, and microsomal glutathione S-transferase 3 (*GSTO1*, *MGST2*, and *MGST3*) are also overexpressed in RTT with a FC = 2.1. For instance, *SOD1* upregulation could be a consequence of increased superoxide production by aberrant activation of complex I and III and the dismutation of superoxide in H_2_O_2_ can explain the increased expression of *CAT* and *PRDX1*. It is likely to believe that the compensatory antioxidant system is not quite sufficient to quench ROS production and this could explain the high OS level present in RTT [22, 23]. For this reason the induction of glutathione S-transferase omega-1 (*GSTO1*) is not surprising. This enzyme is involved in the detoxification mechanisms via conjugation of reduced glutathione (GSH) to oxidativelly modified proteins (carbonyls and 4-HNE PAs). In fact, the GST genes are upregulated in response to OS. In addition, our results showed the upregulation of *MGST2* and *MGST3* which are the microsomal glutathione S-transferases; this data correlates very well with our previous findings where RTT patients showed an increased level of 4-HNE PAs, as *MGST2* is able to also conjugate 4-HNE with GSH [58]. We also observed an increased expression of mRNA for alcohol dehydrogenase 5 (*ADH5*), aldo-keto reductase family 1, member A1 (*AKR1A1*) and aldehyde dehydrogenase 1 family, member A1 (*ALDH1A1*), all enzymes involved in lipid peroxidation products detoxification [59].

### 3.4. Ubiquitin-Proteasome Related Genes in RTT Patients

Our results evidenced also the upregulation of genes related to protein degradation and ubiquitination. In fact, RTT PBMC microarray data revealed increased expression levels of genes associated with protein turnover, such as genes encoding proteasome subunits (*PSMA2*, *PSMA3*, *PSMA5*, *PSMA7*, *PSMB1*, *PSMB10*, *PSMC5*, *PSMC6*, *PSMD6*, and *PSMD9*); furthermore, proteasome maturation protein (*POMP*), involved in proteasome assembly, and genes regulating the activity of the ubiquity ligases (*RBX1*, *UFC1*, *CCNB1IP1*, and *DAXX*) are also up regulated (Table 2), suggesting an increase in cell and protein degradation processes. This could also be a consequence of oxidized proteins and the presence of 4-HNE PAs. This evidence is supported by the observed upregulation of the ubiquitin-conjugating enzyme E2E3 (*UBE2E3*, FC = 1.19) that accepts ubiquitin from the E1 complex and catalyzes its covalent attachment to other proteins. However, this is in contrast with some lines of evidence that link RTT to the downregulation of ubiquitin conjugating enzymes (*UBE3A*) by MeCP2 [60]. Overall the effect of MeCP2 on *UBE3A* regulation is still controversial. In fact, there is even a recent work that did not find any difference in *UBE3A* expression between wild type and a RTT mouse with the mutation R168X [61]. In general, the levels of protein ubiquitination, that is one of the steps to degrade modified proteins, are increased in RTT [24].

### 3.5. Chromatin Folding Related Genes in RTT Patients

In addition several histone related genes (*HIST1H1B*, *HIST1H2AB*, *HIST1H2AI*, *HIST1H2AJ*, *HIST1H2AL*, *HIST1H2BB*, *HIST1H2BH*, *HIST1H2BM*, *HIST1H3B*, *HIST1H3F*, *HIST1H3I*, *HIST1H3J*, *HIST1H4D*, *HIST1H4F*, and *HIST1H4*) were downregulated with a mean FC of −1.28 suggesting a reduced production of proteins necessary to the DNA chromatin assembly.

In general, histone modifications are very dynamic and include acetylation, methylation, isomerization, phosphorylation, and ubiquitination [62]. The combination of such modifications confers enormous variability of cellular signals to environmental stimuli. It is easy to understand that modifications such as histone methylation can display additional complexity since the degree of methylation is very variable (mono-, di-, or trimethylation) [63]. Furthermore, combinations or sequential additions of different histone marks can affect the chromatin organization and subsequently alter the expression of the corresponding target genes.

In our case, several genes related to histone expression were downregulated and this can dramatically affect gene expression. For instance, histone H1 protein binds to linker DNA between nucleosomes forming the macromolecular structure known as the chromatin fiber. Histones H1 are necessary for the condensation of nucleosome chains into higher-order structured fibers. Acts also as a regulator of individual gene transcription through chromatin remodeling, nucleosome spacing, and DNA methylation. We have detected a down-regulation of several H1 subunits ranging from 2- to 3-fold in RTT patients.

### 3.6. Validation of Selected PBMC mRNAs by qPCR Analyses

**Figure 2:**
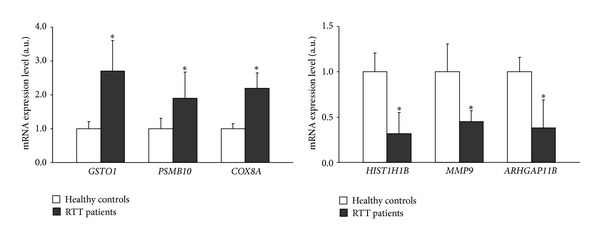
Validation of relative gene expression levels for selected genes using RT-qPCRin PBMC from 12 RTT patients and 7 controls. Results are the means ± SEM of three independent experiments, each analysed in triplicate. *P value < 0.001 versus control (one-way ANOVA followed by Bonferroni's post-test).

Next, we wanted to confirm the differential expression observed for selected mRNAs, on an individual basis, by RT-qPCR. Six genes were selected based on their different patterns of expression (3 up- and 3 downregulated). Assessment of their mRNA expression levels by RT-qPCR accurately reflected those obtained by microarray profiling (Figure 2), thereby confirming the validity of our microarray results. The levels of three mRNAs encoding for glutathione S-transferase omega 1 (*GSTO1*), proteasome (prosome, macropain) subunit, beta type-10 (*PSMB10*), and cytochrome c oxidase subunit VIIIA (*COX8A*) were upregulated in RTT patients by 2.5-, 2- and 2.2-fold, respectively (Figure 2), very similarly to the levels measured by gene array. In contrast, histone cluster 1, H1b (*HIST1H1B*), matrix metallopeptidase 9 (*MMP9*), and Rho GTPase activating protein 11B (*ARHGAP11B*) were downregulated by *∼*50% in RTT patients, as compared to healthy subjects (Figure 2) and also in this case similar expression was detected by gene array analysis.

### 3.7. Conclusion

**Scheme 1:**
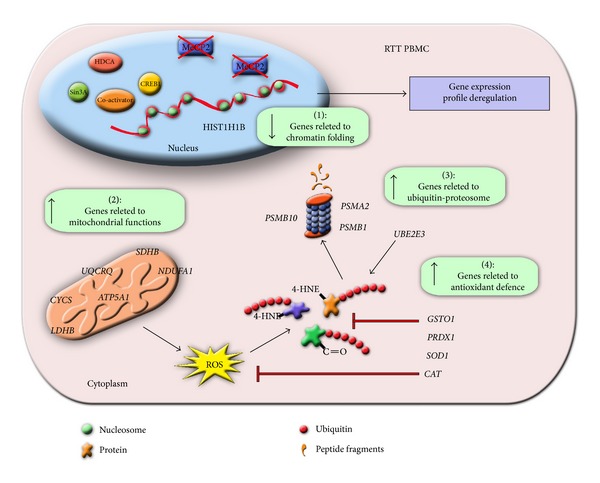
Schematic summary related to the altered gene expression observed in RTT PBMC. MeCP2 in a normal situation binds to several cofactors (Sin3A, CREB1, etc.) to regulate gene transcription. Mutation of MeCP2 (crossed boxes) will affect gene expression leading to a gene profile deregulation. There are mainly 4 gene clusters significantly affected in RTT PBMC, that is, those related to chromatin folding (1), mitochondrial functions (2), ubiquitin-proteasome (3), and antioxidant defence (4) (green boxes). The overexpression of the genes involved in ATP synthesis processes (2) can be interpreted as a possible energy requirement for an increment of cellular protein degradation (3), consequent to increased mitochondrial ROS production and protein oxidation. The increased expression of the “antioxidant cellular defence” genes (4) is the possible compensatory mechanism activated by the cells to quench ROS production and protein oxidation (red arrows).

Our microarray data reveal an altered gene expression profile in RTT lymphomonocytes with the upregulation of genes related to mitochondrial biology and ubiquitin-proteasome proteolytic pathway. In particular, the overexpression of the genes involved in ATP synthesis processes means the tendency of cells to show an altered energy requirement, perhaps to cope with the increased activities of protein degradation. On the other hand, it should be noted that mitochondrion plays essential roles in mediating the production of ROS and these in turn cause damage to proteins, as well as lipids and nucleic acids. To remove damaged molecules, in a kind of vicious circle, increased cellular proteolytic activity requires an extra mitochondrial ATP production with a further ROS generation. This picture is consistent with our previous reports [24], indicating the alteration of redox status in RTT patients, coupled with the increased ubiquitination and degradation of oxidatively modified proteins (Scheme 1).

In conclusion, these findings on transcriptional profiling in RTT patients reveal new molecular mechanisms underlying RTT phenotype, suggesting that mitochondrial-ATP-proteosome are likely to have direct actions on redox balance in RTT syndrome. Furthermore, it confirmed a possible indirect role of OS in pathogenesis and progression of disorder. Thus, RTT should be considered as possible mitochondriopathy.

## Supplementary Material

The complete list of differentially expressed genes, both upregulated and downregulated (FC ± 1), is shown in Supplemental Tables 1 and 2.

## Figures and Tables

**Table 1:** Demographic and genetic data for RTT patients enrolled in study.

No.	Age	Stage	Mutation type	Nucleotide change	Amino acid change
#1	7	3	ETMs		
#2	10	3	missense	c.403A>G	p.K135E
#3	9	4	missense	c.403A>G	p.K135E
#4	9	4	missense	c.455C>G	p.P152R
#5	12	3	missense	c.473C>T	p.T158M
#6	19	3	nonsense	c.763C>T	p.R255X
#7	22	3	frameshift insertion or deletion	c.806_807delG	p.G269fs
#8	7	4	nonsense	c.808C>T	p.R270X
#9	7	3	nonsense	c.808C>T	p.R270X
#10	12	4	nonsense	c.880C>T	p.R294X
#11	6	3	nonsense	c.880C>T	p.R294X
#12	11	3	missense	c.916C>T	p.R306C

ETMs: early truncating mutations.

**Table 2:** Genes upregulated in RTT patients by LIMMA and SAM analyses.

NCBI reference sequence	Gene symbol	Gene name	Molecular function	Biological process	Fold change
NM_004541.3	NDUFA1	NADH dehydrogenase (ubiquinone) 1 alpha subcomplex, 1, 7.5 kDa	NADH dehydrogenase (ubiquinone) activity	Mitochondrial electron transport, NADH to ubiquinone	3.1

NM_006498.2	LGALS2	Lectin, galactoside-binding, soluble, 2	Carbohydrate binding	—	2.8

NM_014302.3	SEC61G	Sec61 gamma subunit	Protein transporter activity	Protein targeting to ER; antigen processing and presentation of exogenous peptide antigen via MHC class I	2.8

NM_001040437.1	C6orf48	Chromosome 6 open reading frame 48	—	—	2.7

NM_001098577.2	RPL31	Ribosomal protein L31	RNA binding; structural constituent of ribosome	Translational elongation; translational initiation; translational termination	2.7

NM_006989.5	RASA4	RAS p21 protein activator 4	GTPase activator activity; phospholipid binding	Intracellular signal transduction; positive regulation of GTPase activity; regulation of small GTPase mediated signal transduction	2.6

NM_000983.3	RPL22	Ribosomal protein L22	RNA binding; heparin binding; structural constituent of ribosome	Alpha-beta T cell differentiation; translational elongation; translational initiation; translational termination	2.6

NM_019059.3	TOMM7	Translocase of outer mitochondrial membrane 7 homolog (yeast)	Protein transmembrane transporter activity	Cellular protein metabolic process; protein import into mitochondrial matrix; protein targeting to mitochondrion	2.6

NM_031157.2	HNRNPA1	Heterogeneous nuclear ribonucleoprotein A1	Nucleotide binding; single-stranded DNA binding; single-stranded RNA binding	RNA export from nucleus; mRNA splicing, via spliceosome; mRNA transport; nuclear import	2.5

NM_001828.5	CLC	Charcot-Leyden crystal galectin	Carbohydrate binding; carboxylesterase activity; lysophospholipase activity	Lipid catabolic process; multicellular organismal development	2.4

NM_032901.3	COX14	Cytochrome c oxidase assembly homolog 14 (S. cerevisiae)	Plays a role in the assembly or stability of the cytochrome c oxidase complex (COX)	Mitochondrial respiratory chain complex IV assembly	2.3

NM_024960.4	PANK2	Pantothenate kinase 2	ATP binding; pantothenate kinase activity	Cell death; coenzyme A biosynthetic process; coenzyme biosynthetic process; pantothenate metabolic process	2.3

NR_002309.1	RPS26P11	Ribosomal protein S26 pseudogene 11	Structural constituent of ribosome	Translation	2.3

NM_005003.2	NDUFAB1	NADH dehydrogenase (ubiquinone) 1, alpha/beta subcomplex, 1, 8 kDa	NADH dehydrogenase (ubiquinone) activity; ACP phosphopantetheine attachment site binding involved in fatty acid biosynthetic process; fatty acid binding; calcium ion binding	Cellular metabolic process; protein lipoylation; small molecule metabolic process; respiratory electron transport chain; fatty acid biosynthetic process; mitochondrial electron transport, NADH to ubiquinone	2.3

NM_152851.2	MS4A6A	Membrane-spanning 4-domains, subfamily A, member 6A	May be involved in signal transduction as a component of a multimeric receptor complex	—	2.3

NM_004269.3	MED27	Mediator complex subunit 27	Transcription coactivator activity	Regulation of transcription from RNA polymerase II promoter; stem cell maintenance; transcription initiation from RNA polymerase II promoter	2.2

NR_015404.1	C12orf47	MAPKAPK5 antisense RNA 1	—	—	2.2

NM_001865.3	COX7A2	Cytochrome c oxidase subunit VIIa polypeptide 2 (liver)	Cytochrome-c oxidase activity; electron carrier activity	Oxidative phosphorylation	2.2

NM_004374.3	COX6C	Cytochrome c oxidase subunit VIc	Cytochrome-c oxidase activity	Respiratory electron transport chain; small molecule metabolic process	2.2

NM_002984.2	CCL4	Chemokine (C-C motif) ligand 4	Chemokine activity	Cell adhesion; cell-cell signaling; chemotaxis; immune response; inflammatory response; positive regulation of calcium ion transport; positive regulation of calcium-mediated signaling; positive regulation of natural killer cell chemotaxis; response to toxic substance; response to virus; signal transduction	2.2

NM_014060.2	MCTS1	Malignant T cell amplified sequence 1	RNA binding	Cell cycle; positive regulation of cell proliferation; regulation of growth; regulation of transcription, DNA-dependent; response to DNA damage stimulus transcription, DNA-dependent	2.1

NM_004832.2	GSTO1	Glutathione S-transferase omega 1	Glutathione dehydrogenase (ascorbate) activity; glutathione transferase activity; methylarsonate reductase activity	L-ascorbic acid metabolic process; glutathione derivative biosynthetic process; negative regulation of ryanodine-sensitive calcium-release channel activity; positive regulation of ryanodine-sensitive calcium-release channel activity; positive regulation of skeletal muscle contraction by regulation of release of sequestered calcium ion; regulation of cardiac muscle contraction by regulation of the release of sequestered calcium ion; xenobiotic catabolic process	2.1

NM_001867.2	COX7C	Cytochrome c oxidase subunit VIIc	Cytochrome-c oxidase activity	Respiratory electron transport chain; small molecule metabolic process	2.1

NM_014206.3	C11orf10	Transmembrane protein 258	—	—	2.1

NM_002413.4	MGST2	Microsomal glutathione S-transferase 2	Enzyme activator activity; glutathione peroxidase activity; glutathione transferase activity; leukotriene-C4 synthase activity	Glutathione biosynthetic process; glutathione derivative biosynthetic process; leukotriene biosynthetic process; positive regulation of catalytic activity; xenobiotic metabolic process	2.1

NM_001124767.1	C3orf78	Small integral membrane protein 4	—	—	2.1

NM_152398.2	OCIAD2	OCIA domain containing 2	—	—	2.1

NM_002488.4	NDUFA2	NADH dehydrogenase (ubiquinone) 1 alpha subcomplex, 2, 8 kDa	NADH dehydrogenase (ubiquinone) activity	Mitochondrial electron transport, NADH to ubiquinone small molecule metabolic process	2.1

NM_004528.3	MGST3	Microsomal glutathione S-transferase 3	Glutathione peroxidase activity; glutathione transferase activity	Glutathione derivative biosynthetic process; lipid metabolic process; signal transduction; small molecule metabolic process xenobiotic metabolic process	2.0

NM_003095.2	SNRPF	Small nuclear ribonucleoprotein polypeptide F	RNA binding	Histone mRNA metabolic process; mRNA 3′-end processing ncRNA metabolic process; spliceosomal snRNP assembly; termination of RNA polymerase II transcription	2.0

NM_005213.3	CSTA	Cystatin A (stefin A)	Cysteine-type endopeptidase inhibitor activity; protein binding, bridging structural molecule activity	Cell-cell adhesion; keratinocyte differentiation; peptide cross-linking	2.0

NM_033318.4	C22orf32	Single-pass membrane protein with aspartate-rich tail 1	—	—	2.0

NM_053035.2	MRPS33	Mitochondrial ribosomal protein S33	Structural constituent of ribosome	Translation	2.0

BC014670.1	LOC147727	Hypothetical protein LOC147727, mRNA (cDNA clone IMAGE: 4864993), partial cds	—	—	2.0

NM_001014.4	RPS10	Ribosomal protein S10	Protein binding	Translational elongation; translational initiation; translational termination; viral transcription	2.0

NM_001130710.1	LSM5	LSM5 homolog, associated U6 small nuclear RNA (S. cerevisiae)	RNA binding	RNA splicing; exonucleolytic nuclear-transcribed mRNA catabolic process involved in deadenylation-dependent decay; mRNA processing	2.0

NM_002801.3	PSMB10	Proteasome (prosome, macropain) subunit, beta type, 10	Threonine-type endopeptidase activity	DNA damage response, signal transduction by p53 class mediator resulting in cell cycle arrest; G1/S transition of mitotic cell cycle; T cell proliferation; anaphase-promoting complex-dependent proteasomal ubiquitin-dependent protein catabolic process; apoptotic process; cell morphogenesis; gene expression; humoral immune response; mRNA metabolic process; negative regulation of apoptotic process; negative regulation of ubiquitin-protein ligase activity involved in mitotic cell cycle; positive regulation of ubiquitin-protein ligase activity involved in mitotic cell cycle; protein polyubiquitination; regulation of cellular amino acid metabolic process; small molecule metabolic process	2.0

NM_014044.5	UNC50	Unc-50 homolog (C. elegans)	RNA binding	Cell surface receptor signaling pathway; protein transport	2.0

NM_032747.3	USMG5	Up-regulated during skeletal muscle growth 5 homolog (mouse)	Plays a critical role in maintaining the ATP synthase population in mitochondria	—	2.0

NM_001001330.2	REEP3	Receptor accessory protein 3	May enhance the cell surface expression of odorant receptors	—	2.0

NM_004074.2	COX8A	Cytochrome c oxidase subunit VIIIA (ubiquitous)	Cytochrome-c oxidase activity	Respiratory electron transport chain; small molecule metabolic process	2.0

NM_002493.4	NDUFB6	NADH dehydrogenase (ubiquinone) 1 beta subcomplex, 6, 17 kDa	NADH dehydrogenase (ubiquinone) activity	Mitochondrial electron transport, NADH to ubiquinone; small molecule metabolic process	2.0

NM_032273.3	TMEM126A	Transmembrane protein 126A	—	Optic nerve development	2.0

“—”: lacking item.

**Table 3:** Genes down-regulated in RTT patients by LIMMA and SAM analyses.

NCBI reference sequence	Gene symbol	Gene name	Molecular function	Biological process	Fold change
NM_170601.4	SIAE	Sialic acid acetylesterase	Sialate O-acetylesterase activity	—	−2.3

NR_002312.1	RPPH1	Ribonuclease P RNA component H1	—	—	−2.2

NM_005322.2	HIST1H1B	Histone cluster 1, H1b	DNA binding	Nucleosome assembly	−1.8

NM_000902.3	MME	Membrane metalloendopeptidase	Metalloendopeptidase activity	Proteolysis	−1.8

NM_032047.4	B3GNT5	UDP-GlcNAc: betaGal beta-1,3-N-acetylglucosaminyltransferase 5	Galactosyltransferase activity	Glycolipid biosynthetic process; posttranslational protein modification	−1.7

NM_003513.2	HIST1H2AB	Histone cluster 1, H2ab	DNA binding	Nucleosome assembly	−1.7

NR_002562.1	SNORD28	Small nucleolar RNA, C/D box 28	—	—	−1.5

NM_004668.2	MGAM	Maltase-glucoamylase (alpha-glucosidase)	Alpha-glucosidase activity; amylase activity	Carbohydrate metabolic process	−1.5

NM_012081.5	ELL2	Elongation factor, RNA polymerase II, 2	—	Regulation of transcription, DNA-dependent	−1.4

NM_021066.2	HIST1H2AJ	Histone cluster 1, H2aj	DNA binding	nucleosome assembly	−1.4

NM_002424.2	MMP8	Matrix metallopeptidase 8 (neutrophil collagenase)	Metalloendopeptidase activity; zinc ion binding	Proteolysis	−1.4

NM_004994.2	MMP9	Matrix metallopeptidase 9 (gelatinase B, 92 kDa, gelatinase, 92 kDa, type IV collagenase)	Collagen binding; metalloendopeptidase activity; zinc ion binding	Collagen catabolic process; extracellular matrix disassembly; positive regulation of apoptotic process; proteolysis	−1.4

NM_003533.2	HIST1H3I	Histone cluster 1, H3i	DNA binding	Nucleosome assembly; regulation of gene silencing	−1.3

NG_000861.4	GK3P	Glycerol kinase 3 pseudogene	ATP binding; glycerol kinase activity	Catabolic process; glycerol metabolic process	−1.3

NR_033423.1	LOC1720	Dihydrofolate reductase pseudogene	—	—	−1.3

NM_003521.2	HIST1H2BM	Histone cluster 1, H2bm	DNA binding	Nucleosome assembly	−13

NM_002417.4	MKI67	Antigen identified by monoclonal antibody Ki-67	ATP binding	DNA metabolic process; cell proliferation; cellular response to heat; meiosis; organ regeneration	−1.2

NM_020406.2	CD177	CD177 molecule	—	Blood coagulation; leukocyte migration	−1.2

NM_001039841.1	ARHGAP11B	Rho GTPase activating protein 11B	GTPase activator activity	Positive regulation of GTPase activity; regulation of small GTPase mediated signal transduction; small GTPase mediated signal transduction	−1.2

NM_001004690.1	OR2M5	Olfactory receptor, family 2, subfamily M, member 5	G-protein coupled receptor activity; olfactory receptor activity	Detection of chemical stimulus involved in sensory perception of smell	−1.2

NM_052966.3	FAM129A	Family with sequence similarity 129, member A	—	Negative regulation of protein phosphorylation; positive regulation of protein phosphorylation; positive regulation of translation; response to endoplasmic reticulum stress	−1.2

NM_001067.3	TOP2A	Topoisomerase (DNA) II alpha 170 kDa	ATP binding; DNA binding, bending; chromatin binding; drug binding; magnesium ion binding; ubiquitin binding	DNA ligation; DNA repair; DNA topological change; DNA-dependent DNA replication; apoptotic chromosome condensation; mitotic cell cycle; phosphatidylinositol-mediated signaling; positive regulation of apoptotic process; positive regulation of retroviral genome replication; positive regulation of transcription from RNA polymerase II promoter; sister chromatid segregation	−1.2

NM_021018.2	HIST1H3F	Histone cluster 1, H3f	DNA binding	S phase; blood coagulation; nucleosome assembly; regulation of gene silencing	−1.2

NM_182707.2	PSG8	Pregnancy specific beta-1-glycoprotein 8	The human pregnancy-specific glycoproteins (PSGs) are a group of molecules that are mainly produced by the placental syncytiotrophoblasts during pregnancy. PSGs comprise a subgroup of the carcinoembryonic antigen (CEA) family, which belongs to the immunoglobulin superfamily.	Female pregnancy	−1.2

NM_003535.2	HIST1H3J	Histone cluster 1, H3j	DNA binding	S phase; blood coagulation; nucleosome assembly; regulation of gene silencing	−1.2

NM_004566.3	PFKFB3	6-Phosphofructo-2-kinase/fructose-2,6-biphosphatase 3	6-Phosphofructo-2-kinase activity; ATP binding; fructose-2,6-bisphosphate 2-phosphatase activity	Fructose metabolic process; glycolysis; small molecule metabolic process	−1.2

NM_016448.2	DTL	Denticleless E3 ubiquitin-protein ligase homolog (Drosophila)	—	DNA replication; G2 DNA damage checkpoint; protein monoubiquitination; protein polyubiquitination; ubiquitin-dependent protein catabolic process	−1.2

NG_001019.5	IGHM	Immunoglobulin heavy constant mu	Antigen binding	Immune response	−1.2

NR_002907.2	SNORA73A	Small nucleolar RNA, H/ACA box 73A	—	—	−1.2

“—”: lacking item.

**Table 4:** GO analysis of genes reported to be deregulated in PBMC of RTT patients.

Term	Description	*P* value	Fold enrichment
Cluster 1			
GO:0022900	Electron transport chain	2.0 × 10^−12^	9.01
GO:0006091	Generation of precursor metabolites and energy	1.7 × 10^−12^	5.06
GO:0045333	Cellular respiration	2.7 × 10^−12^	9.71
GO:0006120	Mitochondrial electron transport, NADH to ubiquinone	4.9 × 10^−12^	16.32
GO:0022904	Respiratory electron transport chain	1.8 × 10^−11^	12.04
GO:0006119	Oxidative phosphorylation	2.0 × 10^−11^	9.17
GO:0042775	Mitochondrial ATP synthesis coupled electron transport	2.0 × 10^−11^	12.99
GO:0042773	ATP synthesis coupled electron transport	2.0 × 10^−11^	12.99
GO:0015980	Energy derivation by oxidation of organic compounds	3.4 × 10^−9^	6.54
GO:0055114	Oxidation reduction	1.5 × 10^−8^	3.01

Cluster 2			
GO:0006626	Protein targeting to mitochondrion	4.3 × 10^−3^	8.81
GO:0070585	Protein localization in mitochondrion	4.3 × 10^−3^	8.81
GO:0045039	Protein import into mitochondrial inner membrane	7.5 × 10^−3^	28.54
GO:0007007	Inner mitochondrial membrane organization	2.8 × 10^−2^	19.03
GO:0006839	Mitochondrial transport	3.1 × 10^−2^	4.96

Cluster 3			
GO:0051436	Negative regulation of ubiquitin-protein ligase activity during mitotic cell cycle	1.8 × 10^−4^	7.24
GO:0051444	Negative regulation of ubiquitin-protein ligase activity	2.1 × 10^−4^	7.03
GO:0051352	Negative regulation of ligase activity	2.1 × 10^−4^	7.03
GO:0051437	Positive regulation of ubiquitin-protein ligase activity during mitotic cell cycle	2.4 × 10^−4^	6.92
GO:0051443	Positive regulation of ubiquitin-protein ligase activity	2.8 × 10^−4^	6.73
GO:0051439	Regulation of ubiquitin-protein ligase activity during mitotic cell cycle	3.0 × 10^−4^	6.63
GO:0051351	Positive regulation of ligase activity	3.7 × 10^−4^	6.45
GO:0051438	Regulation of ubiquitin-protein ligase activity	6.2 × 10^−4^	6.04
GO:0051340	Regulation of ligase activity	8.4 × 10^−4^	5.81
GO:0043086	Negative regulation of catalytic activity	8.4 × 10^−3^	2.78
GO:0044092	Negative regulation of molecular function	9.6 × 10^−3^	2.56

Cluster 4			
GO:0031145	Anaphase-promoting complex-dependent proteasomal ubiquitin-dependent protein catabolic process	1.8 × 10^−4^	7.24
GO:0010498	Proteasomal protein catabolic process	4.4 × 10^−3^	4.62
GO:0043161	Proteasomal ubiquitin-dependent protein catabolic process	4.4 × 10^−3^	4.62

Cluster 5			
GO:0031397	Negative regulation of protein ubiquitination	4.1 × 10^−4^	6.36
GO:0031400	Negative regulation of protein modification process	9.2 × 10^−4^	4.68
GO:0031398	Positive regulation of protein ubiquitination	1.0 × 10^−3^	5.61
GO:0032269	Negative regulation of cellular protein metabolic process	3.2 × 10^−3^	3.58
GO:0031396	Regulation of protein ubiquitination	4.2 × 10^−3^	4.71
GO:0051248	Negative regulation of protein metabolic process	4.4 × 10^−3^	3.43
GO:0031401	Positive regulation of protein modification process	4.1 × 10^−2^	2.98
GO:0032268	Regulation of cellular protein metabolic process	4.7 × 10^−2^	2.08

Cluster 6			
GO:0065003	Macromolecular complex assembly	1.4 × 10^−8^	2.96
GO:0043933	Macromolecular complex subunit organization	9.9 × 10^−8^	2.77
GO:0034622	Cellular macromolecular complex assembly	3.2 × 10^−6^	3.63
GO:0034621	Cellular macromolecular complex subunit organization	2.6 × 10^−5^	3.24
GO:0065004	Protein-DNA complex assembly	1.1 × 10^−4^	6.12
GO:0034728	Nucleosome organization	5.2 × 10^−4^	5.52
GO:0006461	Protein complex assembly	2.1 × 10^−3^	2.37
GO:0070271	Protein complex biogenesis	2.1 × 10^−3^	2.37

Cluster 7			
GO:0006334	Nucleosome assembly	2.4 × 10^−4^	6.12
GO:0031497	Chromatin assembly	3.1 × 10^−4^	5.90
GO:0006323	DNA packaging	8.2 × 10^−4^	4.76
GO:0006333	Chromatin assembly or disassembly	6.0 × 10^−3^	4.04

Cluster 8			
GO:0008380	RNA splicing	5.6 × 10^−6^	3.77
GO:0006396	RNA processing	5.7 × 10^−6^	2.82
GO:0006397	mRNA processing	1.5 × 10^−4^	3.20
GO:0000398	Nuclear mRNA splicing, via spliceosome	2.0 × 10^−4^	4.48
GO:0000377	RNA splicing, via transesterification reactions with bulged adenosine as nucleophile	2.0 × 10^−4^	4.48
GO:0000375	RNA splicing, via transesterification reactions	2.0 × 10^−4^	4.48
GO:0016071	mRNA metabolic process	8.5 × 10^−4^	2.78

Cluster 9			
GO:0006412	Translation	2.6 × 10^−8^	4.01
GO:0006414	Translational elongation	5.4 × 10^−5^	5.93

Cluster 10			
GO:0006367	Transcription initiation from RNA polymerase II promoter	1.1 × 10^−3^	6.30
GO:0006352	Transcription initiation	4.5 × 10^−3^	5.16

Cluster 11			
GO:0006368	RNA elongation from RNA polymerase II promoter	1.1 × 10^−3^	7.13
GO:0006354	RNA elongation	4.5 × 10^−3^	6.71
