# Supplementary material for: Genes Related to Mitochondrial Functions, Protein Degradation, and Chromatin Folding Are Differentially Expressed in Lymphomonocytes of Rett Syndrome Patients
Source: Mediators Inflamm. 2013 Dec 12;2013:137629. doi: 10.1155/2013/137629 (PMC3876710; doi:10.1155/2013/137629)
Supplement: Supplementary file 1 — The complete list of differentially expressed genes, both upregulated and downregulated (FC ± 1), is shown in Supplemental Tables 1 and 2. [file 137629.f1.pdf]

Table 1S. List of all genes resulted up-regulated in RTT *vs* controls by LIMMA and SAM analyses.

NCBI Ref. Seq.	Gene symbol	Gene name	FC
NM_001087.3	AAMP	Angio-associated, migratory cell protein	1.07
NM_181806.2	AASDH	Aminoadipate-semialdehyde dehydrogenase	1.03
NM_032169.4	ACAD11	Acyl-coa dehydrogenase family, member 11	1.01
NM_032360.3	ACBD6	Acyl-coa binding domain containing 6	1.08
NM_022496.4	ACTR6	ARP6 actin-related protein 6 homolog (yeast)	1.43
XM_005268239.1	ACYP1	Acylphosphatase 1, erythrocyte (common) type	1.47
NM_000671.3	ADH5	Alcohol dehydrogenase 5 (class III), chi polypeptide	1.50
NM_018269.3	ADI1	Acireductone dioxygenase 1	1.32
NM_000027.3	AGA	Aspartylglucosaminidase	1.40
NM_006303.3	AIMP2	Aminoacyl trna synthetase complex-interacting multifunctional protein 2	1.06
NM_006066.3	AKR1A1	Aldo-keto reductase family 1, member A1 (aldehyde reductase)	1.23
XM_005251800.1	ALDH1A1	Aldehyde dehydrogenase 1 family, member A1	1.64
NM_013338.4	ALG5	ALG5, dolichyl-phosphate beta-glucosyltransferase	1.76
NM_032306.3	ALKBH7	Alkb, alkylation repair homolog 7 (E. Coli)	1.32
NM_001008221.1	AMY1A	Amylase, alpha 1A (salivary)	1.20
NM_000699.2	AMY2A	Amylase, alpha 2A (pancreatic)	1.50
NM_020978.4	AMY2B	Amylase, alpha 2B (pancreatic)	1.87
NM_016476.10	ANAPC11	Anaphase promoting complex subunit 11	1.81
NM_001097577.2	ANG	Angiogenin, ribonuclease, rnase A family, 5	1.06
NM_023039.4	ANKRA2	Ankyrin repeat, family A (RFXANK-like), 2	1.08
NR_026844.1	ANKRD36BP1	Ankyrin repeat domain 36B pseudogene 1	1.47
NM_021822.3	APOBEC3G	Apolipoprotein B mrna editing enzyme, catalytic polypeptide-like 3G	1.55
NM_004311.3	ARL3	ADP-ribosylation factor-like 3	1.08
NM_032852.3	ATG4C	Autophagy related 4C, cysteine peptidase	1.17
NM_025092.4	ATHL1	ATH1, acid trehalase-like 1 (yeast)	1.09
NM_004046.5	ATP5A1	ATP synthase, H+ transporting, mitochondrial F1 complex, alpha subunit 1, cardiac muscle	1.07
NR_002162.1	ATP5EP2	ATP synthase, H+ transporting, mitochondrial F1 complex, epsilon subunit pseudogene 2	1.02
NM_004889.3	ATP5J2	ATP synthase, H+ transporting, mitochondrial Fo complex, subunit F2	1.22
NM_001697.2	ATP5O	ATP synthase, H+ transporting, mitochondrial F1 complex, O subunit	1.36
NM_015994.3	ATP6V1D	Atpase, H+ transporting, lysosomal 34kda, V1 subunit D	1.24
NM_016311.4	ATPIF1	Atpase inhibitory factor 1	1.04
NM_000712.3	BLVRA	Biliverdin reductase A	1.33
NM_015379.4	BRI3	Brain protein I3	1.23
NM_018462.4	BRK1	BRICK1, SCAR/WAVE actin-nucleating complex subunit	1.14
NM_015415.3	BRP44	Mitochondrial pyruvate carrier 2	1.08
NM_004335.3	BST2	Bone marrow stromal cell antigen 2	1.35
NM_003910.3	BUD31	BUD31 homolog (S. Cerevisiae)	1.54
NM_144591.3	C10orf32	Chromosome 10 open reading frame 32	1.85
NM_014206.3	C11orf10	Transmembrane protein 258	2.10
NM_170746.2	C11orf31	Chromosome 11 open reading frame 31	1.14
NM_020179.2	C11orf75	Single-pass membrane protein with coiled-coil domains 4	1.93
NM_032230.2	C12orf26	Methyltransferase like 25	1.19
NR_015404.1	C12orf47	MAPKAPK5 antisense RNA 1	2.22
NM_138425.2	C12orf57	Chromosome 12 open reading frame 57	1.49
NM_001145199.1	C12orf75	Chromosome 12 open reading frame 75	1.01

NM_016039.2	C14orf166	Chromosome 14 open reading frame 166	1.59
NM_001127393.1	C14orf2	Chromosome 14 open reading frame 2	1.37
NM_020233.4	C17orf48	ADP-ribose/CDP-alcohol diphosphatase, manganese-dependent	1.20
NR_027160.1	C17orf76-AS1	C17orf76 antisense RNA 1	1.01
NM_001012985.2	C1orf31	Cytochrome c oxidase assembly factor 6 homolog (S. Cerevisiae)	1.43
NM_024579.3	C1orf54	Chromosome 1 open reading frame 54	1.69
NM_020317.3	C1orf63	Chromosome 1 open reading frame 63	1.04
NM_033318.4	C22orf32	Single-pass membrane protein with aspartate-rich tail 1	2.02
NM_001124767.1	C3orf78	Small integral membrane protein 4	2.09
NM_017867.2	C4orf27	Chromosome 4 open reading frame 27	1.56
NM_001145432.1	C4orf52	Small integral membrane protein 20	1.73
NM_022483.4	C5orf28	Chromosome 5 open reading frame 28	1.02
NM_001014279.2	C5orf39	Annexin A2 receptor	1.75
NM_024941.3	C5orf44	Trafficking protein particle complex 13	1.14
NM_021243.2	C6orf115	ABRA C-terminal like	1.35
NM_001008739.1	C6orf226	Chromosome 6 open reading frame 226	1.19
NM_001040437.1	C6orf48	Chromosome 6 open reading frame 48	2.73
NM_018341.2	C6orf70	ER membrane-associated RNA degradation	1.04
NM_001008395.2	C7orf59	Late endosomal/lysosomal adaptor, MAPK and MTOR activator 4	1.84
NM_138436.3	C8orf40	Small integral membrane protein 19	1.49
NM_018465.3	C9orf46	Plasminogen receptor, C-terminal lysine transmembrane protein	1.42
NM_001743.4	CALM2	Calmodulin 2 (phosphorylase kinase, delta)	1.40
NM_001745.3	CAMLG	Calcium modulating ligand	1.30
NM_001747.3	CAPG	Capping protein (actin filament), gelsolin-like	1.18
NM_001752.3	CAT	Catalase	1.14
NM_018491.3	CBWD1	COBW domain containing 1	1.21
NM_201453.2	CBWD3	COBW domain containing 3	1.30
XM_005272748.1	CBWD5	COBW domain containing 5	1.69
NM_138493.2	CCDC167	Coiled-coil domain containing 167	1.54
NM_199342.3	CCDC23	Coiled-coil domain containing 23	1.14
NM_015439.2	CCDC28A	Coiled-coil domain containing 28A	1.02
NM_001017928.2	CCDC58	Coiled-coil domain containing 58	1.09
NM_015933.3	CCDC72	Translation machinery associated 7 homolog (S. Cerevisiae)	1.69
NM_002984.2	CCL4	Chemokine (C-C motif) ligand 4	2.15
NM_001001435.2	CCL4L1	Chemokine (C-C motif) ligand 4-like 1	1.03
NM_021178.4	CCNB1IP1	Cyclin B1 interacting protein 1, E3 ubiquitin protein ligase	1.13
XM_005264083.1	CCT4	Chaperonin containing TCP1, subunit 4 (delta)	1.17
NM_001762.3	CCT6A	Chaperonin containing TCP1, subunit 6A (zeta 1)	1.07
NM_004244.5	CD163	CD163 molecule	1.57
NM_001765.2	CD1C	CD1c molecule	1.84
NM_001242.4	CD27	CD27 molecule	1.04
NM_000732.4	CD3D	CD3d molecule, delta (CD3-TCR complex)	1.83
XM_005269238.1	CD63	CD63 molecule	1.50
NM_001783.3	CD79A	CD79a molecule, immunoglobulin-associated alpha	1.51
XM_005257858.1	CD79B	CD79b molecule, immunoglobulin-associated beta	1.13
NM_176096.2	CDK5RAP3	CDK5 regulatory subunit associated protein 3	1.04
XM_005266966.1	CENPW	Centromere protein W	1.36
NM_006324.2	CFDP1	Craniofacial development protein 1	1.01
XM_058325.3	CHCHD1	Coiled-coil-helix-coiled-coil-helix domain containing 1	1.71
NM_016139.2	CHCHD2	Coiled-coil-helix-coiled-coil-helix domain containing 2	1.25

NM_001280.2	CIRBP	Cold inducible RNA binding protein	1.48
NM_001828.5	CLC	Charcot-Leyden crystal galectin	2.38
NM_006344.2	CLEC10A	C-type lectin domain family 10, member A	1.19
NM_138337.5	CLEC12A	C-type lectin domain family 12, member A	1.45
NM_005127.2	CLEC2B	C-type lectin domain family 2, member B	1.14
NM_194450.2	CLEC4A	C-type lectin domain family 4, member A	1.51
NM_182523.1	CMC1	COX assembly mitochondrial protein 1 homolog (S. Cerevisiae)	1.34
NM_020188.3	CMC2	COX assembly mitochondrial protein 2 homolog (S. Cerevisiae)	1.19
NM_014184.3	CNIH4	Cornichon family AMPA receptor auxiliary protein 4	1.37
NM_001008215.2	COA5	Cytochrome c oxidase assembly factor 5	1.41
NM_152516.2	COMMD1	Copper metabolism (Murr1) domain containing 1	1.04
NM_012071.3	COMMD3	COMM domain containing 3	1.53
XM_085023.6	COMMD6	COMM domain containing 6	1.63
NM_017845.3	COMMD8	COMM domain containing 8	1.35
NM_001258006.1	COPS4	COP9 signalosome subunit 4	1.13
XM_005251137.1	COPS5	COP9 signalosome subunit 5	1.37
NM_032901.3	COX14	Cytochrome c oxidase assembly homolog 14 (S. Cerevisiae)	2.34
NM_016468.6	COX16	COX16 cytochrome c oxidase assembly homolog (S. Cerevisiae)	1.25
NM_005694.1	COX17	COX17 cytochrome c oxidase copper chaperone	1.10
NM_001862.2	COX5B	Cytochrome c oxidase subunit Vb	1.64
NM_001863.4	COX6B1	Cytochrome c oxidase subunit vib polypeptide 1 (ubiquitous)	1.24
NM_004374.3	COX6C	Cytochrome c oxidase subunit vic	2.17
NM_001865.3	COX7A2	Cytochrome c oxidase subunit viia polypeptide 2 (liver)	2.20
NM_001866.2	COX7B	Cytochrome c oxidase subunit viib	1.63
NM_001867.2	COX7C	Cytochrome c oxidase subunit viic	2.11
NM_004074.2	COX8A	Cytochrome c oxidase subunit VIIIA (ubiquitous)	1.98
NM_019029.2	CPVL	Carboxypeptidase, vitellogenic-like	1.38
NM_175918.3	CRIPAK	Cysteine-rich PAK1 inhibitor	1.67
NM_000099.2	CST3	Cystatin C	1.19
NM_005213.3	CSTA	Cystatin A (stefin A)	2.02
NM_016403.3	CWC15	CWC15 spliceosome-associated protein homolog (S. Cerevisiae)	1.78
NM_007022.3	CYB561D2	Cytochrome b561 family, member D2	1.05
NM_148923.3	CYB5A	Cytochrome b5 type A (microsomal)	1.16
NM_018947.5	CYCS	Cytochrome c, somatic	1.09
XM_005248858.1	DAXX	Death-domain associated protein	1.03
XM_005248974.1	DDAH2	Dimethylarginine dimethylaminohydrolase 2	1.08
XM_005250808.1	DECR1	2,4-dienoyl coa reductase 1, mitochondrial	1.03
NM_006519.2	DYNLT1	Dynein, light chain, Tctex-type 1	1.41
NM_006520.2	DYNLT3	Dynein, light chain, Tctex-type 3	1.10
NM_032565.4	EBPL	Emopamil binding protein-like	1.14
XM_005267047.1	ECHDC1	Enoyl coa hydratase domain containing 1	1.11
NM_001037663.1	EEF1B2	Eukaryotic translation elongation factor 1 beta 2	1.16
NM_005875.2	EIF1B	Eukaryotic translation initiation factor 1B	1.37
NM_004846.2	EIF4E2	Eukaryotic translation initiation factor 4E family member 2	1.63
NM_006331.7	EMG1	EMG1 N1-specific pseudouridine methyltransferase	1.19
NM_001425.2	EMP3	Epithelial membrane protein 3	1.40
NR_015370.2	EPB41L4A-AS1	EPB41L4A antisense RNA 1	1.12
XM_005266278.1	ESD	Esterase D	1.24
NM_000126.3	ETFA	Electron-transfer-flavoprotein, alpha polypeptide	1.13
NM_015004.3	EXOSC7	Exosome component 7	1.35

NM_031452.3	FAM103A1	Family with sequence similarity 103, member A1	1.46
NM_031213.3	FAM108A1	Abhydrolase domain containing 17A	1.01
NM_014367.3	FAM162A	Family with sequence similarity 162, member A	1.21
NM_058182.4	FAM165B	Small integral membrane protein 11	1.15
NM_152274.4	FAM58A	Family with sequence similarity 58, member A	1.02
XM_005258656.1	FCGRT	Fc fragment of igg, receptor, transporter, alpha	1.30
NM_002013.3	FKBP3	FK506 binding protein 3, 25kda	1.16
NM_002027.2	FNTA	Farnesyltransferase, CAAX box, alpha	1.03
NM_007285.6	GABARAPL2	GABA(A) receptor-associated protein-like 2	1.11
NR_003191.1	GGTA1P	Glycoprotein, alpha-galactosyltransferase 1 pseudogene	1.51
NM_015660.2	GIMAP2	Gtpase, IMAP family member 2	1.18
NM_015710.4	GLTSCR2	Glioma tumor suppressor candidate region gene 2	1.15
NM_006613.3	GRAP	GRB2-related adaptor protein	1.23
NM_004832.2	GSTO1	Glutathione S-transferase omega 1	2.11
NM_004492.2	GTF2A2	General transcription factor IIA, 2, 12kda	1.12
NM_207118.2	GTF2H5	General transcription factor IIH, polypeptide 5	1.13
NM_002104.2	GZMK	Granzyme K (granzyme 3; tryptase II)	1.00
NM_002106.3	H2AFZ	H2A histone family, member Z	1.60
NM_002107.4	H3F3A	H3 histone, family 3A	1.00
NM_000518.4	HBB	Hemoglobin, beta	1.40
NM_006402.2	HBXIP	Late endosomal/lysosomal adaptor, MAPK and MTOR activator 5	1.09
XM_005254328.1	HDC	Histidine decarboxylase	1.04
NM_015987.4	HEBP1	Heme binding protein 1	1.34
NM_006120.3	HLA-DMA	Major histocompatibility complex, class II, DM alpha	1.12
NM_022555.3	HLA-DRB3	Major histocompatibility complex, class II, DR beta 3	1.10
NM_021983.4	HLA-DRB4	Major histocompatibility complex, class II, DR beta 4	1.03
NM_001130688.1	HMGB2	High mobility group box 2	1.16
NM_004965.6	HMGN1	High mobility group nucleosome binding domain 1	1.07
NM_004242.3	HMGN3	High mobility group nucleosomal binding domain 3	1.34
NM_031157.2	HNRNPA1	Heterogeneous nuclear ribonucleoprotein A1	2.48
XM_005269748.1	HNRNPH3	Heterogeneous nuclear ribonucleoprotein H3 (2H9)	1.05
NM_004493.2	HSD17B10	Hydroxysteroid (17-beta) dehydrogenase 10	1.14
NM_016245.3	HSD17B11	Hydroxysteroid (17-beta) dehydrogenase 11	1.12
NM_014234.4	HSD17B8	Hydroxysteroid (17-beta) dehydrogenase 8	1.26
NM_016126.2	HSPB11	Heat shock protein family B (small), member 11	1.93
NM_002157.2	HSPE1	Heat shock 10kda protein 1 (chaperonin 10)	1.20
NM_001098521.1	HTATIP2	HIV-1 Tat interactive protein 2, 30kda	1.23
NM_032036.2	IFI27L2	Interferon, alpha-inducible protein 27-like 2	1.03
NM_004221.4	IL32	Interleukin 32	1.06
XM_005274431.1	IL3RA	Interleukin 3 receptor, alpha (low affinity)	1.34
NM_001564.2	ING2	Inhibitor of growth family, member 2	1.06
NM_004763.3	ITGB1BP1	Integrin beta 1 binding protein 1	1.10
NM_004867.4	ITM2A	Integral membrane protein 2A	1.43
NM_001267623.1	ITPA	Inosine triphosphatase (nucleoside triphosphate pyrophosphatase)	1.44
XM_005250233.1	KLHDC10	Kelch domain containing 10	1.86
NM_002258.2	KLRB1	Killer cell lectin-like receptor subfamily B, member 1	1.41
NM_173852.3	KRTCAP2	Keratinocyte associated protein 2	1.12
NM_001032998.1	KYNU	Kynureninase	1.47
NM_014017.3	LAMTOR2	Late endosomal/lysosomal adaptor, MAPK and MTOR activator 2	1.32
NM_002300.6	LDHB	Lactate dehydrogenase B	1.32

NM_002305.3	LGALS1	Lectin, galactoside-binding, soluble, 1	1.30
NM_006498.2	LGALS2	Lectin, galactoside-binding, soluble, 2	2.80
XR_042051.1	LINC00152	Long intergenic non-protein coding RNA 152	1.05
XR_241187.1	LINC00339	Long intergenic non-protein coding RNA 339	1.12
BC014670.1	LOC147727	Hypothetical protein LOC147727, mrna (cdna clone IMAGE:4864993), partial cds	2.01
BC065765.1	LOC401397	Hypothetical LOC401397, mrna (cdna clone IMAGE:4699553)	1.37
NM_005767.5	LPAR6	Lysophosphatidic acid receptor 6	1.56
NM_018334.4	LRRN3	Leucine rich repeat neuronal 3	1.01
NM_014463.2	LSM3	LSM3 homolog, U6 small nuclear RNA associated (S. Cerevisiae)	1.29
NM_001130710.1	LSM5	LSM5 homolog, U6 small nuclear RNA associated (S. Cerevisiae)	2.00
NM_007080.2	LSM6	LSM6 homolog, U6 small nuclear RNA associated (S. Cerevisiae)	1.58
NM_002341.1	LTB	Lymphotoxin beta (TNF superfamily, member 3)	1.47
NM_014060.2	MCTS1	Malignant T cell amplified sequence 1	2.13
NM_138476.3	MDP1	Magnesium-dependent phosphatase 1	1.34
NM_004269.3	MED27	Mediator complex subunit 27	2.24
NM_014166.3	MED4	Mediator complex subunit 4	1.39
NM_004270.4	MED7	Mediator complex subunit 7	1.19
NM_001410.2	MEGF8	Multiple EGF-like-domains 8	1.10
NM_019852.3	METTL3	Methyltransferase like 3	1.27
NM_032889.4	MFSD5	Major facilitator superfamily domain containing 5	1.67
NM_002413.4	MGST2	Microsomal glutathione S-transferase 2	2.10
NM_004528.3	MGST3	Microsomal glutathione S-transferase 3	2.04
NR_029519.1	MIR103A2	Microrna 103a-2	1.10
NR_029683.1	MIR142	Microrna 142	1.81
NM_138798.1	MITD1	MIT, microtubule interacting and transport, domain containing 1	1.33
NM_002432.1	MNDA	Myeloid cell nuclear differentiation antigen	1.57
NM_023075.5	MPPE1	Metallophosphoesterase 1	1.09
NM_014078.5	MRPL13	Mitochondrial ribosomal protein L13	1.37
NM_017971.3	MRPL20	Mitochondrial ribosomal protein L20	1.41
NM_181512.1	MRPL21	Mitochondrial ribosomal protein L21	1.00
NM_004891.3	MRPL33	Mitochondrial ribosomal protein L33	1.91
NM_017446.3	MRPL39	Mitochondrial ribosomal protein L39	1.22
NM_016497.3	MRPL51	Mitochondrial ribosomal protein L51	1.12
NM_180982.2	MRPL52	Mitochondrial ribosomal protein L52	1.32
NM_053050.4	MRPL53	Mitochondrial ribosomal protein L53	1.09
NM_016070.3	MRPS23	Mitochondrial ribosomal protein S23	1.37
NM_022497.3	MRPS25	Mitochondrial ribosomal protein S25	1.09
NM_016640.3	MRPS30	Mitochondrial ribosomal protein S30	1.27
NM_005830.3	MRPS31	Mitochondrial ribosomal protein S31	1.48
NM_053035.2	MRPS33	Mitochondrial ribosomal protein S33	2.02
XM_005274395.1	MS4A1	Membrane-spanning 4-domains, subfamily A, member 1	1.16
NM_001031666.1	MS4A3	Membrane-spanning 4-domains, subfamily A, member 3 (hematopoietic cell-specific)	1.20
XM_005274177.1	MS4A6A	Membrane-spanning 4-domains, subfamily A, member 6A	2.25
NM_016100.4	NAA20	N(alpha)-acetyltransferase 20, natb catalytic subunit	1.44
NM_016200.4	NAA38	N(alpha)-acetyltransferase 38, natc auxiliary subunit	1.16
NM_004541.3	NDUFA1	NADH dehydrogenase (ubiquinone) 1 alpha subcomplex, 1, 7.5kda	3.11
NM_002488.4	NDUFA2	NADH dehydrogenase (ubiquinone) 1 alpha subcomplex, 2, 8kda	2.06
NM_014222.2	NDUFA8	NADH dehydrogenase (ubiquinone) 1 alpha subcomplex, 8, 19kda	1.20
NM_005002.4	NDUFA9	NADH dehydrogenase (ubiquinone) 1 alpha subcomplex, 9, 39kda	1.51

NM_005003.2	NDUFAB1	NADH dehydrogenase (ubiquinone) 1, alpha/beta subcomplex, 1, 8kda	2.26
NM_004548.2	NDUFB10	NADH dehydrogenase (ubiquinone) 1 beta subcomplex, 10, 22kda	1.45
NM_004546.2	NDUFB2	NADH dehydrogenase (ubiquinone) 1 beta subcomplex, 2, 8kda	1.41
NM_004547.5	NDUFB4	NADH dehydrogenase (ubiquinone) 1 beta subcomplex, 4, 15kda	1.44
NM_002493.4	NDUFB6	NADH dehydrogenase (ubiquinone) 1 beta subcomplex, 6, 17kda	1.98
NM_005005.2	NDUFB9	NADH dehydrogenase (ubiquinone) 1 beta subcomplex, 9, 22kda	1.30
NM_002494.3	NDUFC1	NADH dehydrogenase (ubiquinone) 1, subcomplex unknown, 1, 6kda	1.34
NM_004549.5	NDUFC2	NADH dehydrogenase (ubiquinone) 1, subcomplex unknown, 2, 14.5kda	1.42
NM_002495.2	NDUFS4	NADH dehydrogenase (ubiquinone) Fe-S protein 4, 18kda (NADH-coenzyme Q reductase)	1.51
NM_004552.2	NDUFS5	NADH dehydrogenase (ubiquinone) Fe-S protein 5, 15kda (NADH-coenzyme Q reductase)	1.40
NM_004553.4	NDUFS6	NADH dehydrogenase (ubiquinone) Fe-S protein 6, 13kda (NADH-coenzyme Q reductase)	1.47
NM_021074.4	NDUFV2	NADH dehydrogenase (ubiquinone) flavoprotein 2, 24kda	1.52
NM_006156.2	NEDD8	Neural precursor cell expressed, developmentally down-regulated 8	1.50
NM_015700.3	NFU1	NFU1 iron-sulfur cluster scaffold homolog (S. Cerevisiae)	1.20
XM_005265316.1	NGLY1	N-glycanase 1	1.07
NM_015469.1	NIPSNAP3A	Nipsnap homolog 3A (C. Elegans)	1.16
XM_005258964.1	NOSIP	Nitric oxide synthase interacting protein	1.43
NM_006545.4	NPRL2	Nitrogen permease regulator-like 2 (S. Cerevisiae)	1.02
NM_145080.3	NSMCE1	Non-SMC element 1 homolog (S. Cerevisiae)	1.49
NM_032324.1	NTPCR	Nucleoside-triphosphatase, cancer-related	1.04
NM_145266.4	NUDCD2	Nudc domain containing 2	1.15
NM_000274.3	OAT	Ornithine aminotransferase	1.10
NM_152398.2	OCIAD2	OCIA domain containing 2	2.07
NM_024960.4	PANK2	Pantothenate kinase 2	2.27
NM_003631.2	PARG	Poly (ADP-ribose) glycohydrolase	1.22
NM_018622.5	PARL	Presenilin associated, rhomboid-like	1.01
NM_000925.3	PDHB	Pyruvate dehydrogenase (lipoamide) beta	1.33
NM_002567.2	PEBP1	Phosphatidylethanolamine binding protein 1	1.25
XM_005265696.1	PF4	Platelet factor 4	1.54
NM_002623.3	PFDN4	Prefoldin subunit 4	1.38
NM_014260.3	PFDN6	Prefoldin subunit 6	1.09
NM_017916.2	PIH1D1	PIH1 domain containing 1	1.24
NM_001001852.3	PIM3	Pim-3 oncogene	1.25
NM_006223.3	PIN4	Protein (peptidylprolyl cis/trans isomerase) NIMA-interacting, 4 (parvulin)	1.54
NM_004279.2	PMPCB	Peptidase (mitochondrial processing) beta	1.04
NM_019896.2	POLE4	Polymerase (DNA-directed), epsilon 4, accessory subunit	1.19
NM_021974.3	POLR2F	Polymerase (RNA) II (DNA directed) polypeptide F	1.08
NM_006232.3	POLR2H	Polymerase (RNA) II (DNA directed) polypeptide H	1.06
NM_006233.4	POLR2I	Polymerase (RNA) II (DNA directed) polypeptide I, 14.5kda	1.12
NM_006234.4	POLR2J	Polymerase (RNA) II (DNA directed) polypeptide J, 13.3kda	1.17
NM_015932.5	POMP	Proteasome maturation protein	1.14
NM_015918.3	POP5	Processing of precursor 5, ribonuclease P/MRP subunit (S. Cerevisiae)	1.23
NM_006112.3	PPIE	Peptidylprolyl isomerase E (cyclophilin E)	1.26
NM_002712.2	PPP1R7	Protein phosphatase 1, regulatory subunit 7	1.19
NM_017917.2	PPP2R3C	Protein phosphatase 2, regulatory subunit B", gamma	1.03
NM_015342.3	PPWD1	Peptidylprolyl isomerase domain and WD repeat containing 1	1.14
NM_181697.2	PRDX1	Peroxiredoxin 1	1.12
NM_007173.4	PRSS23	Protease, serine, 23	1.09

NM_002787.4	PSMA2	Proteasome (prosome, macropain) subunit, alpha type, 2	1.81
NM_002788.3	PSMA3	Proteasome (prosome, macropain) subunit, alpha type, 3	1.49
NM_002790.3	PSMA5	Proteasome (prosome, macropain) subunit, alpha type, 5	1.06
NM_002792.3	PSMA7	Proteasome (prosome, macropain) subunit, alpha type, 7	1.57
NM_002793.3	PSMB1	Proteasome (prosome, macropain) subunit, beta type, 1	1.43
NM_002801.3	PSMB10	Proteasome (prosome, macropain) subunit, beta type, 10	1.99
NM_002805.5	PSMC5	Proteasome (prosome, macropain) 26S subunit, atpase, 5	1.15
NM_002806.3	PSMC6	Proteasome (prosome, macropain) 26S subunit, atpase, 6	1.80
NM_014814.2	PSMD6	Proteasome (prosome, macropain) 26S subunit, non-atpase, 6	1.05
NM_002813.5	PSMD9	Proteasome (prosome, macropain) 26S subunit, non-atpase, 9	1.18
XM_005264248.1	QPCT	Glutaminyl-peptide cyclotransferase	1.47
NM_130781.2	RAB24	RAB24, member RAS oncogene family	1.19
NM_006834.3	RAB32	RAB32, member RAS oncogene family	1.15
NM_004582.3	RABGGTB	Rab geranylgeranyltransferase, beta subunit	1.31
NM_006989.5	RASA4	RAS p21 protein activator 4	2.60
NM_005105.4	RBM8A	RNA binding motif protein 8A	1.37
NM_052960.2	RBP7	Retinol binding protein 7, cellular	1.77
NM_014248.3	RBX1	Ring-box 1, E3 ubiquitin protein ligase	1.40
NM_001001330.2	REEP3	Receptor accessory protein 3	1.98
NM_001033002.3	RPAIN	RPA interacting protein	1.01
NM_007104.4	RPL10A	Ribosomal protein l10a	1.73
NM_000977.3	RPL13	Ribosomal protein L13	1.20
NM_002948.3	RPL15	Ribosomal protein L15	1.47
NM_000983.3	RPL22	Ribosomal protein L22	2.56
XM_114317.7	RPL22L1	Ribosomal protein L22-like 1	1.57
NM_000987.3	RPL26	Ribosomal protein L26	1.10
NM_001098577.2	RPL31	Ribosomal protein L31	2.68
NM_000994.3	RPL32	Ribosomal protein L32	1.08
NM_052969.1	RPL39L	Ribosomal protein L39-like	1.17
XM_005249271.1	RPS10	Ribosomal protein S10	2.01
NG_011176.1	RPS14P3	Ribosomal protein S14 pseudogene 3	1.23
NG_010375.1	RPS18P9	Ribosomal protein S18 pseudogene 9	1.17
NM_001028.2	RPS25	Ribosomal protein S25	1.57
NM_001029.3	RPS26	Ribosomal protein S26	1.46
NR_002309.1	RPS26P11	Ribosomal protein S26 pseudogene 11	2.27
NM_001030001.2	RPS29	Ribosomal protein S29	1.50
NM_001009.3	RPS5	Ribosomal protein S5	1.89
NM_001011.3	RPS7	Ribosomal protein S7	1.26
NM_015485.4	RWDD3	RWD domain containing 3	1.22
NM_002961.2	S100A4	S100 calcium binding protein A4	1.94
NM_014624.3	S100A6	S100 calcium binding protein A6	1.51
NM_002965.3	S100A9	S100 calcium binding protein A9	1.74
NM_033082.3	SARNP	SAP domain containing ribonucleoprotein	1.39
NM_133491.3	SAT2	Spermi dine/spermine N1-acetyltransferase family member 2	1.05
NM_024041.3	SCNM1	Sodium channel modifier 1	1.11
NM_005138.2	SCO2	SCO2 cytochrome c oxidase assembly protein	1.20
XM_005263170.1	SCOC	Short coiled-coil protein	1.23
NM_006923.3	SDF2	Stromal cell-derived factor 2	1.06
NM_022044.2	SDF2L1	Stromal cell-derived factor 2-like 1	1.27
NR_003265.3	SDHAP2	Succinate dehydrogenase complex, subunit A, flavoprotein pseudogene 2	1.12

NM_003000.2	SDHB	Succinate dehydrogenase complex, subunit B, iron sulfur (Ip)	1.20
NM_014302.3	SEC61G	Sec61 gamma subunit	2.76
NM_001122752.1	SERPINI1	Serpin peptidase inhibitor, clade I (neuroserpin), member 1	1.27
NM_016047.3	SF3B14	Splicing factor 3B, 14 kda subunit (SF3B14), mrna	1.41
NM_031287.2	SF3B5	Splicing factor 3b, subunit 5, 10kda	1.14
NM_145169.1	SFT2D1	SFT2 domain containing 1	1.91
NM_006304.1	SHFM1	Split hand/foot malformation (ectrodactyly) type 1	1.66
NM_001126105.2	SLC7A7	Solute carrier family 7 (amino acid transporter light chain, y+L system), member 7	1.32
NM_005901.5	SMAD2	SMAD family member 2	1.31
NR_024127.1	SNHG12	Small nucleolar RNA host gene 12 (non-protein coding)	1.55
NR_002576.1	SNORA21	Small nucleolar RNA, H/ACA box 21	1.10
NR_002987.1	SNORA61	Small nucleolar RNA, H/ACA box 61	1.06
NR_004380.1	SNORD104	Small nucleolar RNA, C/D box 104	1.18
NR_000018.1	SNORD35A	Small nucleolar RNA, C/D box 35A	1.18
NR_000016.1	SNORD36C	Small nucleolar RNA, C/D box 36C	1.36
NR_000007.1	SNORD73A	Small nucleolar RNA, C/D box 73A	1.08
NR_003940.1	SNORD80	Small nucleolar RNA, C/D box 80	1.12
NM_006938.2	SNRPD1	Small nuclear ribonucleoprotein D1 polypeptide 16kda	1.03
NM_003094.2	SNRPE	Small nuclear ribonucleoprotein polypeptide E	1.71
NM_003095.2	SNRPF	Small nuclear ribonucleoprotein polypeptide F	2.04
NM_003096.2	SNRPG	Small nuclear ribonucleoprotein polypeptide G	1.40
NM_003097.3	SNRPN	Small nuclear ribonucleoprotein polypeptide N	1.56
NM_014748.3	SNX17	Sorting nexin 17	1.06
NM_000454.4	SOD1	Superoxide dismutase 1, soluble	1.60
NM_004890.2	SPAG7	Sperm associated antigen 7	1.31
NM_014752.2	SPCS2	Signal peptidase complex subunit 2 homolog (S. Cerevisiae)	1.54
NM_001166103.1	SPINT2	Serine peptidase inhibitor, Kunitz type, 2	1.28
NM_138288.3	SPTSSA	Serine palmitoyltransferase, small subunit A	1.07
NM_003130.3	SRI	Sorcin	1.64
NM_001031684.2	SRSF7	Serine/arginine-rich splicing factor 7	1.51
NM_003143.2	SSBP1	Single-stranded DNA binding protein 1, mitochondrial	1.58
XM_005249880.1	STARD3NL	STARD3 N-terminal like	1.06
NM_004853.2	STX8	Syntaxin 8	1.33
NM_016086.2	STYXL1	Serine/threonine/tyrosine interacting-like 1	1.09
NM_003172.3	SURF1	Surfeit 1	1.15
NM_015484.4	SYF2	SYF2 pre-mrna-splicing factor	1.49
NM_016283.4	TAF9	TAF9 RNA polymerase II, TATA box binding protein (TBP)-associated factor, 32kda	1.23
NM_032026.3	TATDN1	Tatd dnase domain containing 1	1.64
NM_004607.2	TBCA	Tubulin folding cofactor A	1.64
NM_005648.3	TCEB1	Transcription elongation factor B (SIII), polypeptide 1 (15kda, elongin C)	1.24
NM_152773.4	TCTEX1D2	Tctex1 domain containing 2	1.49
NM_138501.5	TECR	Trans-2,3-enoyl-coa reductase	1.11
NM_025075.2	THOC7	THO complex 7 homolog (Drosophila)	1.30
NM_012456.2	TIMM10	Translocase of inner mitochondrial membrane 10 homolog (yeast)	1.02
NM_012458.3	TIMM13	Translocase of inner mitochondrial membrane 13 homolog (yeast)	1.12
NM_006335.2	TIMM17A	Translocase of inner mitochondrial membrane 17 homolog A (yeast)	1.35
NM_012459.2	TIMM8B	Translocase of inner mitochondrial membrane 8 homolog B (yeast)	1.04
NM_012460.2	TIMM9	Translocase of inner mitochondrial membrane 9 homolog (yeast)	1.02
NM_018447.2	TMEM111	ER membrane protein complex subunit 3	1.12

NM_032273.3	TMEM126A	Transmembrane protein 126A	1.95
NM_018480.4	TMEM126B	Transmembrane protein 126B	1.41
NM_032928.3	TMEM141	Transmembrane protein 141	1.68
NM_032635.3	TMEM147	Transmembrane protein 147	1.34
NM_014051.3	TMEM14A	Transmembrane protein 14A	1.75
NM_016462.3	TMEM14C	Transmembrane protein 14C	1.09
NM_053045.1	TMEM203	Transmembrane protein 203	1.82
NM_198536.2	TMEM205	Transmembrane protein 205	1.16
NM_014187.3	TMEM208	Transmembrane protein 208	1.31
NM_032936.3	TMEM60	Transmembrane protein 60	1.72
NM_015959.3	TMX2	Thioredoxin-related transmembrane protein 2	1.09
NM_019059.3	TOMM7	Translocase of outer mitochondrial membrane 7 homolog (yeast)	2.56
NM_016058.2	TPRKB	TP53RK binding protein	1.62
NM_024108.2	TRAPPC6A	Trafficking protein particle complex 6A	1.30
NM_017910.3	TRMT61B	Trna methyltransferase 61 homolog B (S. Cerevisiae)	1.04
NM_007311.3	TSPO	Translocator protein (18kda)	1.31
NM_001270483.1	TST	Thiosulfate sulfurtransferase (rhodanese)	1.14
NM_001008237.1	TTC32	Tetratricopeptide repeat domain 32	1.78
NM_005783.3	TXNDC9	Thioredoxin domain containing 9	1.03
NM_006701.2	TXNL4A	Thioredoxin-like 4A	1.28
NM_144987.2	U2AF1L4	U2 small nuclear RNA auxiliary factor 1-like 4	1.02
NM_006357.3	UBE2E3	Ubiquitin-conjugating enzyme E2E 3	1.19
NM_016406.3	UFC1	Ubiquitin-fold modifier conjugating enzyme 1	1.33
NM_014044.5	UNC50	Unc-50 homolog (C. Elegans)	1.99
NM_006003.2	UQCRFS1	Ubiquinol-cytochrome c reductase, Rieske iron-sulfur polypeptide 1	1.51
NM_006004.2	UQCRH	Ubiquinol-cytochrome c reductase hinge protein	1.80
NM_014402.4	UQCRQ	Ubiquinol-cytochrome c reductase, complex III subunit VII, 9.5kda	1.54
NM_032747.3	USMG5	Up-regulated during skeletal muscle growth 5 homolog (mouse)	1.99
NM_003761.4	VAMP8	Vesicle-associated membrane protein 8	1.57
NM_198481.3	VSTM1	V-set and transmembrane domain containing 1	1.35
NM_025234.1	WDR61	WD repeat domain 61	1.60
NM_018081.2	WRAP53	WD repeat containing, antisense to TP53	1.05
NM_058181.1	YBEY	Ybey metallopeptidase (putative)	1.14
NM_024699.2	ZFAND1	Zinc finger, AN1-type domain 1	1.01
NM_006963.4	ZMAT2	Zinc finger, matrin-type 2	1.14
NM_006963.4	ZNF22	Zinc finger protein 22	1.02
NM_021994.2	ZNF277	Zinc finger protein 277	1.11
NM_033414.2	ZNF622	Zinc finger protein 622	1.23
NR_003604.2	ZNFX1-AS1	ZNFX1 antisense RNA 1	1.16

Table 2S. List of all genes resulted down-regulated in RTT *vs* controls by LIMMA and SAM analyses.

NCBI Ref. Seq.	Gene symbol	Gene name	FC
NM_170601.4	SIAE	Sialic acid acetylesterase	- 2.3
NR_002312.1	RPPH1	Ribonuclease P RNA component H1	- 2.2
NM_005322.2	HIST1H1B	Histone cluster 1, h1b	- 1.8
NM_000902.3	MME	Membrane metallo-endopeptidase	- 1.8
NM_032047.4	B3GNT5	UDP-glcnac:betagal beta-1,3-N-acetylglucosaminyltransferase 5	- 1.7
NM_003513.2	HIST1H2AB	Histone cluster 1, h2ab	- 1.7
NR_002562.1	SNORD28	Small nucleolar RNA, C/D box 28	- 1.5
NM_004668.2	MGAM	Maltase-glucoamylase (alpha-glucosidase)	- 1.5
NM_012081.5	ELL2	Elongation factor, RNA polymerase II, 2	- 1.4
NM_021066.2	HIST1H2AJ	Histone cluster 1, h2aj	- 1.4
NM_002424.2	MMP8	Matrix metallopeptidase 8 (neutrophil collagenase)	- 1.4
NM_004994.2	MMP9	Matrix metallopeptidase 9 (gelatinase B, 92kda gelatinase, 92kda type IV collagenase)	- 1.4
NM_003533.2	HIST1H3I	Histone cluster 1, h3i	- 1.3
NG_000861.4	GK3P	Glycerol kinase 3 pseudogene	- 1.3
NR_033423.1	LOC1720	Dihydrofolate reductase pseudogene	- 1.3
NM_003521.2	HIST1H2BM	Histone cluster 1, h2bm	- 1.3
NM_001530.3	HIF1A	Hypoxia inducible factor 1, alpha subunit (basic helix-loop-helix transcription factor)	- 1.3
NM_002417.4	MKI67	Antigen identified by monoclonal antibody Ki-67	- 1.2
NM_020406.2	CD177	CD177 molecule	- 1.2
NM_001039841.1	ARHGAP11B	Rho gtpase activating protein 11B	- 1.2
NM_001004690.1	OR2M5	Olfactory receptor, family 2, subfamily M, member 5	- 1.2
NM_052966.3	FAM129A	Family with sequence similarity 129, member A	- 1.2
NM_001067.3	TOP2A	Topoisomerase (DNA) II alpha 170kda	- 1.2
NM_021018.2	HIST1H3F	Histone cluster 1, h3f	- 1.2
NM_182707.2	PSG8	Pregnancy specific beta-1-glycoprotein 8	- 1.2
NM_003535.2	HIST1H3J	Histone cluster 1, h3j	- 1.2
NM_004566.3	PFKFB3	6-phosphofructo-2-kinase/fructose-2,6-biphosphatase 3	- 1.2
NM_016448.2	DTL	Denticleless E3 ubiquitin protein ligase homolog (Drosophila)	- 1.2
NG_001019.5	IGHM	Immunoglobulin heavy constant mu	- 1.2
NR_002907.2	SNORA73A	Small nucleolar RNA, H/ACA box 73A	- 1.2
NM_017813.4	IMPAD1	Inositol monophosphatase domain containing 1	- 1.1
NM_021062.2	HIST1H2BB	Histone cluster 1, h2bb	- 1.1
NM_003540.3	HIST1H4F	Histone cluster 1, h4f	- 1.1
NM_017548.4	CDV3	CDV3 homolog (mouse)	- 1.1
NM_000045.3	ARG1	Arginase 1	- 1.1
NR_026823.1	FAM138D	Family with sequence similarity 138, member D	- 1.1
NM_015295.2	SMCHD1	Structural maintenance of chromosomes flexible hinge domain containing 1	- 1.1
NM_003524.2	HIST1H2BH	Histone cluster 1, h2bh	- 1.1
NR_003302.1	SNORD115-10	Small nucleolar RNA, C/D box 115-10	- 1.1
NM_006418.4	OLFM4	Olfactomedin 4	- 1.1
NM_003539.3	HIST1H4D	Histone cluster 1, h4d	- 1.1
NM_003546.2	HIST1H4L	Histone cluster 1, h4l	- 1.1
NM_014791.3	MELK	Maternal embryonic leucine zipper kinase	- 1.1
NG_000834.1	IGKC	Immunoglobulin kappa constant	- 1.1
NM_000715.3	C4BPA	Complement component 4 binding protein, alpha	- 1.1
NM_003509.2	HIST1H2AI	Histone cluster 1, h2ai	- 1.1
NM_003511.2	HIST1H2AL	Histone cluster 1, h2al	- 1.1

NR_003137.2	RNU4-2	RNA, U4 small nuclear 2	- 1.1
NR_002937.2	IGBP1P1	Immunoglobulin (CD79A) binding protein 1 pseudogene 1	- 1.0
NM_001274.5	CHEK1	Checkpoint kinase 1	- 1.0
NM_016836.3	RBMS1	RNA binding motif, single stranded interacting protein 1	- 1.0
NM_005645.3	TAF13	TAF13 RNA polymerase II, TATA box binding protein (TBP)-associated factor, 18kda	- 1.0
